# Velocity range-based reward shaping technique for effective map-less navigation with LiDAR sensor and deep reinforcement learning

**DOI:** 10.3389/fnbot.2023.1210442

**Published:** 2023-09-06

**Authors:** HyeokSoo Lee, Jongpil Jeong

**Affiliations:** ^1^Department of Smart Factory Convergence, AI Factory Lab, Sungkyunkwan University, Suwon, Republic of Korea; ^2^Research & Development Team, THiRA-UTECH Co., Ltd., Seoul, Republic of Korea

**Keywords:** autonomous mobile robot, deep reinforcement learning, continuous action, map-less navigation, SLAM, LiDAR, reward shaping

## Abstract

In recent years, sensor components similar to human sensory functions have been rapidly developed in the hardware field, enabling the acquisition of information at a level beyond that of humans, and in the software field, artificial intelligence technology has been utilized to enable cognitive abilities and decision-making such as prediction, analysis, and judgment. These changes are being utilized in various industries and fields. In particular, new hardware and software technologies are being rapidly applied to robotics products, showing a level of performance and completeness that was previously unimaginable. In this paper, we researched the topic of establishing an optimal path plan for autonomous driving using LiDAR sensors and deep reinforcement learning in a workplace without map and grid coordinates for mobile robots, which are widely used in logistics and manufacturing sites. For this purpose, we reviewed the hardware configuration of mobile robots capable of autonomous driving, checked the characteristics of the main core sensors, and investigated the core technologies of autonomous driving. In addition, we reviewed the appropriate deep reinforcement learning algorithm to realize the autonomous driving of mobile robots, defined a deep neural network for autonomous driving data conversion, and defined a reward function for path planning. The contents investigated in this paper were built into a simulation environment to verify the autonomous path planning through experiment, and an additional reward technique “Velocity Range-based Evaluation Method” was proposed for further improvement of performance indicators required in the real field, and the effectiveness was verified. The simulation environment and detailed results of experiments are described in this paper, and it is expected as guidance and reference research for applying these technologies in the field.

## 1. Introduction

The Fourth Industrial Revolution, also known as Industry 4.0, refers to a change in the industrial environment that maximizes intelligence, automation, and connectivity through the convergence of more advanced information and communication technologies and existing industrial sectors. Industry 4.0 defines nine core technology areas, and one of them is autonomous robots (Kovács et al., 2018). Along with the emergence of the 4th industrial revolution and the associated digital transformation, the use of robots in many industrial fields is diversifying and increasing rapidly. From 2016 to 2021, the global robot market grew by 11% annually; from 2022 to 2025, it is expected to grow by 7–10% annually (IFR, 2022). Moreover, the recent rapid drop in the birth rate has led to a shortage of manpower, and the use of robots is a very attractive countermeasure to address such manpower shortages. In terms of industry, robots are widely used in medical/health care, professional cleaning, transportation/logistics, and hospitality, and their use in 2021 grew by 23–85% (IFR, 2022). Among them, the application of robots in transportation/logistics is particularly noteworthy.

Mobile robots have become more popular in the marketplace thanks to Amazon, the world's largest e-commerce company. In 2012, Amazon acquired Kiva Robotics, a mobile robotics startup, and deployed mobile robots in its large warehouses, reducing operating costs by about 20% and reducing logistics turnaround time from 60 to 75 min to 15 min. Amazon's case has raised market expectations that robots can be used in workplaces and public facilities to achieve low cost and high efficiency. In addition, Amazon is currently conducting research and development on various types of delivery robots using mobile robots and autonomous vehicles suitable for long-distance delivery. The intelligence and sophistication of robots are developing rapidly, and their use will evolve and accelerate from simply replacing repetitive tasks to skilled and specialized areas.

Mobile robots can be divided into Automated Guided Vehicles (AGVs) and Autonomous Mobile Robots (AMRs). An AGV can only move along a fixed path. This means that it is necessary to have information about the moving space for such a robot to be able to move. The robot is controlled and moved with a magnetic tape or a Quick Response (QR) code attached to the floor (Giuseppe et al., 2021). When using these methods, the environment for controlling the mobile robot must be built on the floor so that the robot can recognize the path to move, which requires a lot of money and time (Giuseppe et al., 2021). Because of these disadvantages, AGVs are rapidly being eclipsed by the AMR method using autonomous driving technology. Recently, it has been confirmed that the market which in 2017 was a 1 billion-dollar market will rapidly grow in 2022 into a 7 billion-dollar market (IFR, 2022).

The evolution of mobile robots is shifting from AMRs operating in warehouses or factories to delivery robots delivering or shipping items outside of buildings. To achieve this, mobile robots need to be advanced to the point where they can drive unmanned on roads with limited space, and delivery robots need to actively utilize various core technologies of autonomous vehicles. In addition, standards and laws are being actively established for autonomous vehicles, and delivery robots must consider these autonomous driving laws and standards. For example, the J3016 SAE Levels of Driving Automation standard released by SAE (Society of Automotive Engineers) International in 2018 is a typical example of a standard related to autonomous vehicles. SAE J3016 defines six levels of automation and establishes the initial regulatory framework and safety design standards for autonomous driving systems in road vehicles. The US Department of Transportation (USDot) has adopted J3016 in its federal autonomous vehicle policy, and J3016 is increasingly recognized as a global standard (SAE International Website, 2021).

There are various skills involved in making a mobile robot. Among various autonomous driving technologies, the most important technology is Simultaneous Localization and Mapping (SLAM) technology, which collects and moves the robot's current real-time and surrounding environment information in real-time through the robot's attached sensors (Giuseppe et al., 2021). Hardware reference guides and examples of mobile robots for autonomous driving were reviewed, as were the principles and key technologies of SLAM. LiDAR sensors were mostly recommended in the hardware reference guide and in the example of mobile robots for autonomous driving, and multiple experiments have decided to use LiDAR sensors (Zhang et al., 2019; Chen et al., 2021; Juan, 2021). Another important technology is artificial intelligence, which can be used to learn path planning. Robots have recently been developing to a level at which they are capable of making judgments and decisions in much more complex environments and situations. In such contexts, a robot must be able to consider uncertainty and flexibly respond to changes in the environment. For a robot to properly operate in a new environment, it must expand and improve its intelligence from the information it initially acquired, and this can be achieved through a method termed learning. Therefore, the learning of robots requires observing the environment as an input and performing actions corresponding to changes in the environment as an output (Zhang et al., 2020; Adithya et al., 2021; Ibarz et al., 2021; Pavlos et al., 2021; Liu et al., 2022; Raj and Kos, 2022). These characteristics are very similar to the reinforcement learning method in which state information is received from the environment, reward information is received from the reward function, and corresponding actions are performed.

Therefore, studies related to the path planning learning of mobile robots through reinforcement learning have been conducted in the past (Lee and Jeong, 2021; Lee et al., 2022), and reinforcement learning technology is applied as an extension in this paper. The papers reviewed when preparing for this paper were cases in which autonomous driving of mobile robots was implemented using LiDAR sensors and deep reinforcement learning (Lei et al., 2017; Grando et al., 2021). The reward method of reinforcement learning in these papers defined reward values for the case of arriving at the target position, the case of collision, and the case of moving to the target position (Lei et al., 2017; Zhang et al., 2020; Adithya et al., 2021; Pavlos et al., 2021). In particular, in cases where it is necessary to move a robot to the target position, methods of giving a fixed reward value or a variable reward value based on distance were used to define the reward value (Lei et al., 2017; Zhang et al., 2020; Adithya et al., 2021; Pavlos et al., 2021). In this study, we propose a reward method for path planning with improved performance and stability by considering other factors beyond reward value for distance when moving to the target location and verifying the results through the experiments.

This paper expects to make the following contributions:

1. This paper explains the basic principles and characteristics of AMR and SLAM, and it compares the operating principles and characteristics of key sensors to help elucidate how the sensor technology can be used for SLAM.
2. We explain the concept of global and local path planning and explain how SLAM and reinforcement learning techniques serve to implement map-less navigation path planning. We also explain the process used to identify the reinforcement learning type suitable for the path planning characteristics and determine the reinforcement learning algorithm to be used in the experiment.
3. This paper explains the architecture for the experiment, the simulation environment, the software design of reinforcement learning, the proposed reward technique for performance improvement, and the parameter values to be used in the experiment. It also visualizes the results of the experiments and explains the analysis results and meanings of the experiments. In this process, the effect of the proposed reward technique is verified.
4. The basic configuration to implement AMR using LiDAR sensor and deep reinforcement learning is comprehensively presented, and a practical reward technique for performance improvement in path planning through reinforcement learning is proposed and verified.

This paper proceeds as follows: Section 2 reviews the mechanical characteristics of AMR, principles of autonomous driving, hardware configuration, main principles and characteristics of SLAM, comparison between LiDAR and RADAR sensors, path planning using SLAM and reinforcement learning, etc. It also explains the reinforcement learning types suitable for autonomous driving and the reinforcement learning algorithms to be used in the experiments. Section 3 presents the proposed system architecture, software design with deep reinforcement learning, and reward function with the proposed technique. Section 4 details the simulation environment, the parameters to be used in the experiments, and the experimental results. Finally, Section 5 offers the final conclusions, opinions, and future research directions.

## 2. Materials

### 2.1. Autonomous driving and autonomous robots

In this paper, we researched the optimal path planning of an autonomous mobile robot with a LiDAR sensor in a warehouse environment via deep reinforcement learning. Most people think of autonomous vehicles when they hear the word autonomous driving, but autonomous driving technology is also of great interest in the robotics industry. Typical types of autonomous robots that utilize autonomous driving technology are autonomous mobile robots and delivery robots. The mobile robot market is growing rapidly and the related technologies are mature to commercialize. On the other hand, delivery robots are in their early stages, and many things need to be prepared and considered for actual commercialization and practical application.

Mobile robots and delivery robots have a lot in common. Delivery robots transport goods in the same way as mobile robots, but the main difference is that they do not transport goods in a single place such as a warehouse or factory, but include outdoor driving to remote locations. Autonomous driving is the most important capability for delivery robots, and location estimation technology and object/environment recognition technology are very important. From the perspective of autonomous driving technology, indoor autonomous driving technology using LiDAR has reached the level of practical application, but outdoor driving technology has many problems to be solved and requires significant institutionalization and regulatory adjustment for practical application (West, 2016). Currently, many technology companies, large enterprises, and startups are conducting research and development to develop delivery robots, and some delivery robots have been launched on a trial basis or are undergoing early experiments.

In this article, we studied the indoor navigation of mobile robots, but we will also briefly look at major examples of products related to delivery robots from the perspective of autonomous driving.

- Starship Technologies, an Estonian startup, has been operating a delivery service for goods and groceries using delivery robots in six locations in the United States and the United Kingdom since 2019. Starship's delivery robots utilize cameras, GPS, and inertial sensors for autonomous driving and do not use LiDAR sensors to reduce the unit cost of products. Object detection and movement area recognition were replaced by Convolution Neural Networks (CNN) based image recognition technology with cameras (Starship Technologies Website, 2019).
- In 2019, Amazon in the United States also introduced a delivery service demonstration using a delivery robot named Scout. Similar to Starship, it did not use LiDAR sensors but utilized cameras and Convolution Neural Networks (CNN) based image recognition technology (Vincent, 2019).
- Marble, a Silicon Valley startup in the United States, implemented autonomous driving in a similar way to existing indoor autonomous robots and outdoor autonomous vehicles. It utilized 3D precise maps and 3D LiDAR sensors acquired in advance to estimate the global position and used LiDAR sensors to detect obstacles and recognize sidewalks. Marble's delivery robot can operate in a more complex urban environment than Starship's delivery robot and has higher stability through multiple sensors. However, there are disadvantages such as the need to build a precise spatial map in advance, the increase in embedded parts, and the high cost of robot products due to expensive parts (Marble debuts its autonomous food-delivery robots in partnership with Yelp, 2017).

### 2.2. Kinematic modeling of autonomous mobile robot

**Figure 1:**
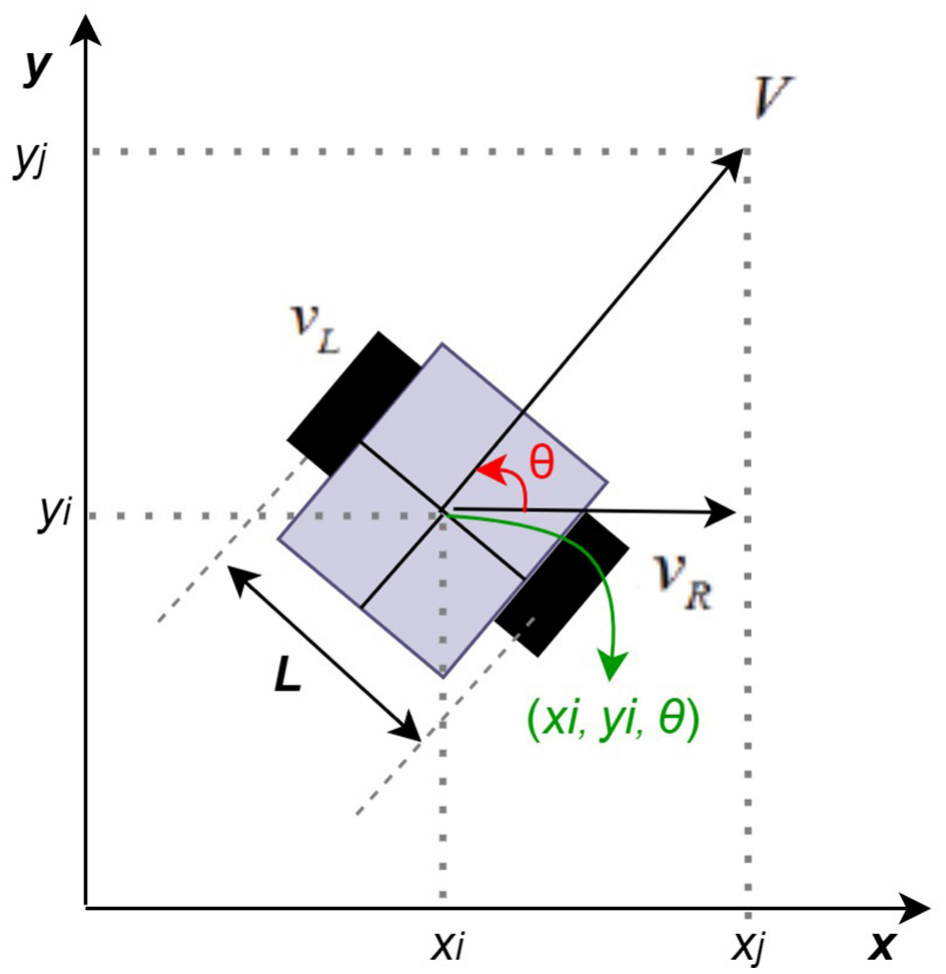
Basic kinematic example of AMR (Kim, 2002; Han et al., 2014; Baek, 2021).

To understand the movement path and autonomous driving characteristics of a mobile robot, it is first necessary to elucidate the kinematic composition of the mobile robot. The mobile robot shown in Figure 1 is an example of the simplest type of mobile robot, which has a structure in which two wheels are located on the same axis, with each wheel is independently controlled by a motor.

The speed of the right shift is *v*_*R*_, the speed of the left shift is *v*_*L*_, and the distance between the two shifts is *L*. The mobile robot's velocity *V* and counterclockwise angular velocity ω are determined by the velocity of the two wheels, and they are respectively expressed by Equations 1, 2 (Kim, 2002; Han et al., 2014; Yu et al., 2020).


(1) $$V=\frac{v_{R}+v_{L}}{2}$$

(2) $$\omega =\frac{v_{R}-v_{L}}{L}$$

Figure 1 shows that the position of the robot in coordinates is *x*_*i*_*(t), y*_*i*_*(t)*. When the position and direction are expressed as vectors, it can be expressed as Equation 3 (Kim, 2002; Han et al., 2014).


(3) $$P\;={\left[\begin{array}{ccc} x_{i} & y_{i} & \theta \end{array}\right]}^{T}$$

The change in the center coordinates of the mobile robot is the same as in Equation 4, and it can be defined as: ẋ_*i*_(*t*) = cosθ·*V*, ẏ_*i*_(*t*) = sinθ·*V*, $\overset{•}{\theta }\left(t\right)=\omega$, so the movement of the mobile robot can be defined as shown in Equation 5 (Kim, 2002; Han et al., 2014).


(4) $$P\;={\left[\begin{array}{ccc} x_{i} & y_{i} & \theta \end{array}\right]}^{T}$$

(5) $$\left[\begin{array}{c} {\dot{x}}_{i}\\ {\dot{y}}_{i}\\ \dot{\theta } \end{array}\right]=\left[\begin{array}{cc} cos\;\theta & 0\\ sin\;\theta & 0\\ 0 & 1 \end{array}\right]\left[\begin{array}{c} V\\ \omega \end{array}\right]$$

### 2.3. Hardware of the autonomous mobile robot

The hardware configuration of the autonomous mobile robot consists of a control module, a driver module, a power module, and a sensor module (Zhang et al., 2019; Liu et al., 2022). The power module consists of a battery and a power control system. The control module which is the main part of the hardware consists of a Single Board Computer (SBC) and a Microcontroller (MCU) (Zhang et al., 2019; Liu et al., 2022). The SBC and MCU work interdependently; the SBC is the center of the decision-making system and implements various control systems. Meanwhile, the MCU can control peripheral devices and some sensors, and a Real-Time Operating System (RTOS) is embedded in the MCU (Zhang et al., 2019). The sensor module is necessary for the path search of the mobile robot, and as can be seen in the hardware configuration example below, it is composed of a LiDAR sensor and an Inertial Measurement Unit (IMU) (Zhang et al., 2019). The mobile robot can check the surrounding environment by using data from the LiDAR sensor and the SLAM algorithm and make targeted movements. The driver module is a motor controller for driving (Zhang et al., 2019).

**Figure 2:**
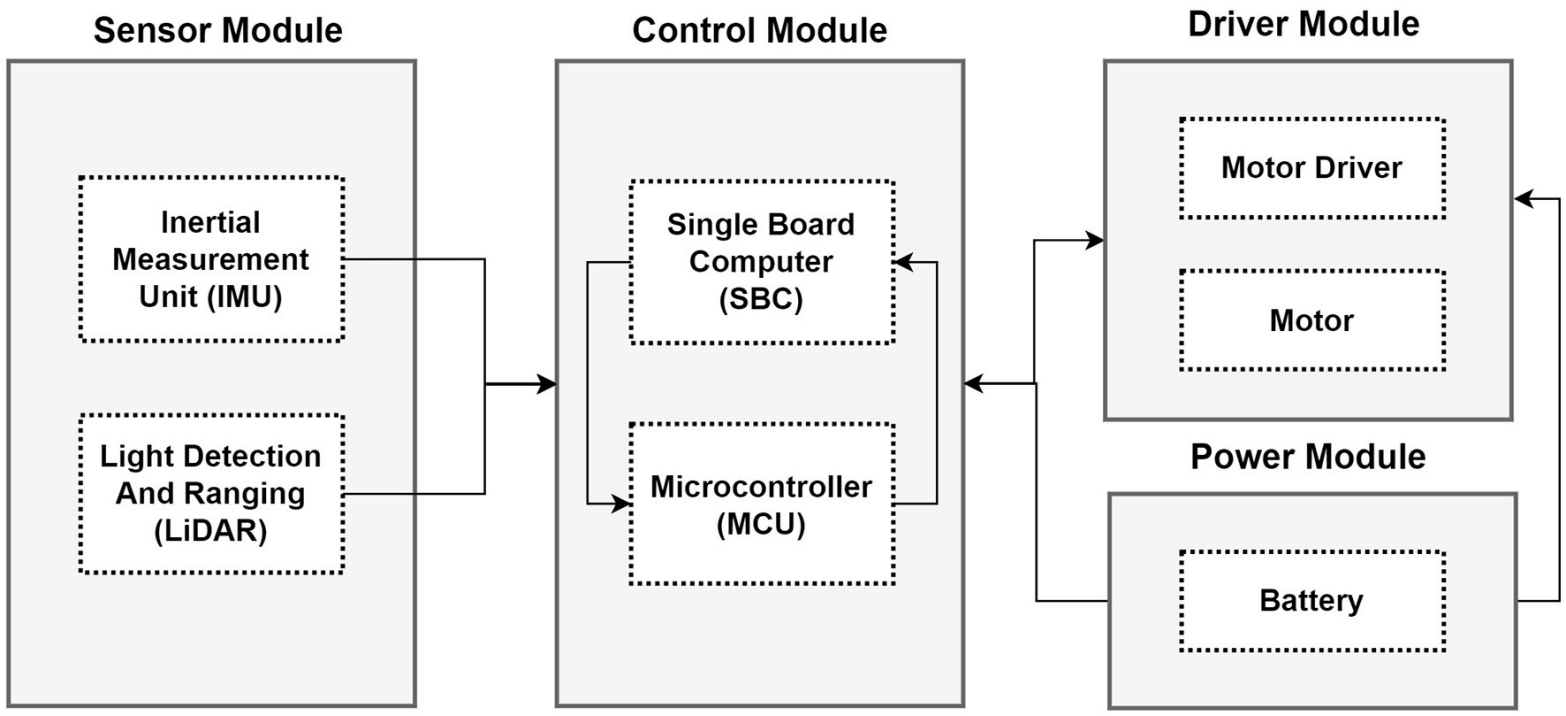
Hardware structure of an autonomous mobile robot (Murat et al., 2017; Zhang et al., 2019; Liu et al., 2022).

Figure 2 shows an example of the hardware configuration of an autonomous mobile robot.

### 2.4. Basic principles and features of SLAM

The most important concepts in the autonomous driving of mobile robots are mapping and localization (Liu, 2021). Mapping is the process of making a map of a predefined environment so that a mobile robot can accurately move (Liu, 2021). Specifically, changes in the environment may occur due to changes in external environmental conditions in the process of searching for a new path. Localization is the process of locating a mobile robot in the environment (Liu, 2021). To find the movement path, it is very important to find the place where the mobile robot starts moving. These steps can be most simply performed using a Global Positioning System (GPS) sensor (Liu, 2021). Using the GPS sensor has the advantage of being inexpensive, but it can also lead to measurement errors. Therefore, higher accuracy can be secured if the configuration uses a sensor such as LiDAR.

Simultaneous Localization and Mapping (SLAM) is a method of mapping and localization at the same time, and when a destination is given, the robot uses sensor or image data to estimate its own location and then moves to the destination based on this technology (Liu, 2021). To perform these SLAM functions, it is necessary to track the robot's location information, surrounding landmark information, and distance information from the target point. Recently, the most frequently utilized sensors for AMR to acquire surrounding information are cameras, Light Detection and Ranging (LiDAR) sensors, and Laser Detection and Ranging (LADAR) sensors. SLAM technology can be divided into Visual and Laser SLAM (Chan et al., 2018; Liu, 2021).

#### 2.4.1. Visual SLAM vs. Laser SLAM

Visual SLAM extracts image features and estimates obstacle maps based on geometry theory (Chan et al., 2018; Baek, 2021). Oriented Fast and Rotated BRIEF SLAM (ORB-SLAM) is a representative Visual SLAM method, and it involves using the ORB algorithm to find feature points at high speed. This method finds the location of the map and the robot by estimating changes in multi-frame images using a camera and image sensor, then calculating the distance to an object using the accumulated changes (Chan et al., 2018; Baek, 2021). This method is a feature point-based method that can be used to estimate location by extracting feature points from an image and estimating movement. However, while this method has the advantage of being relatively inexpensive and simple to apply in terms of cost, it has the disadvantage of being sensitive to the environment along with being weak against noise by extracting information from images (Chan et al., 2018; Baek, 2021). Due to the large amount of data involved, the computation is burdensome, and the accuracy and performance may both be limited in practice (Chan et al., 2018; Baek, 2021).

Laser SLAM was commercialized earlier than the Visual SLAM method, so it is more technologically mature and has a relatively large number of use cases. A typical example is the GMapping method, which directly constructs an obstacle map of the environment based on the results of high-density laser distance measurement (Chan et al., 2018; Liu, 2021). Laser SLAM methods generally use LiDAR sensors frequently. Although the cost is relatively higher than Visual SLAM, it has many use cases due to its high reliability and accuracy. However, using high-density laser sensors is burdensome because it takes a lot of time to construct landmarks and maps, and the performance is highly dependent on sensor accuracy (Chan et al., 2018; Liu, 2021). Recently, the Real-Time Appearance-Based Mapping (RTAB–MAP) method has also emerged as a Visual SLAM method that utilizes a LiDAR sensor. This method has the advantage of being able to detect a range that cannot be detected using a LiDAR sensor (Chan et al., 2018; Liu, 2021).

#### 2.4.2. Light detection and ranging vs. laser detection and ranging

The most widely used sensors for autonomous driving are LiDAR and RADAR sensors. These image sensors are responsible for visual functions in autonomous driving. The two sensors have almost the same purposes, but they work in different ways: LiDAR sensors use lasers whereas RADAR sensors use radio waves. A LiDAR sensor emits high-powered laser pulses, measures the characteristics of the return signal after the laser strikes the target, and then determines the distance, shape, and position between objects. RADAR sensors work similarly to Li-DAR sensors, except they use radio waves instead of lasers; they emit radio waves that hit objects, then they utilize the returned data to grasp information.

The pros and cons of these two sensors are:

- Precision: As explained for the LiDAR sensor, the LiDAR sensor uses a laser. The characteristics of the laser are strong linearity, so when it hits an object and returns data there is almost no distortion and the error range is very small, thus allowing for precise observation (Ryde and Hillier, 2009). LiDAR sensors can also measure distance, width, and height, so they have the advantage of recognizing them in 3D. The LiDAR sensor is easy to map in 3D because a lot of information can be confirmed by increasing the number of channels and splitting and emitting (Ryde and Hillier, 2009). Meanwhile, RADAR sensors can grasp information, such as the distance between objects, speed, and direction, but they have limitations in their ability to grasp the shapes of objects. Therefore, LiDAR sensors are widely used in autonomous driving.
- Cost: In general, LIDAR sensors cost much more than RADAR sensors (Wevolver, 2021). Although LiDAR sensors have many advantages in autonomous driving, the sensor prices are still the biggest reason why manufacturers do not readily decide to use them. An increasing number of companies are trying to reduce prices by reducing the observation range of the existing LiDAR sensor and lowering the performance of the part.
- Sensitivity to the external environment: In the case of RADAR sensors, there is almost no change in changes in the external environment (Ryde and Hillier, 2009; Wevolver, 2021). For example, the same performance and accuracy can be maintained without a significant change even in environments such as rain, snow, and fog (Ryde and Hillier, 2009; Wevolver, 2021). In the case of radio waves, little is absorbed when they hit an object compared with the light, such as a laser. This is also the reason why RADAR is preferred in the case of combat equipment. Comparatively, LiDAR sensors are more sensitive to these changes than RADAR sensors, and they can thus be more affected.

### 2.5. Path planning using SLAM and reinforcement learning

Path planning to find the optimal route is very important for mobile robots. In general, path planning can be divided into global path planning and local path planning. If the mobile robot has environmental information, global path planning can be used; if there is no environmental information, local path planning should be used (Zhu and Zhang, 2021). In other words, global path planning provides all information about the environment before the mobile robot moves, then utilizes this information for movement. Local path planning does not require any information before the mobile robot starts to move, and the robot should use sensors to detect objects and check the information to create a local path plan (Zhu and Zhang, 2021).

**Figure 3:**
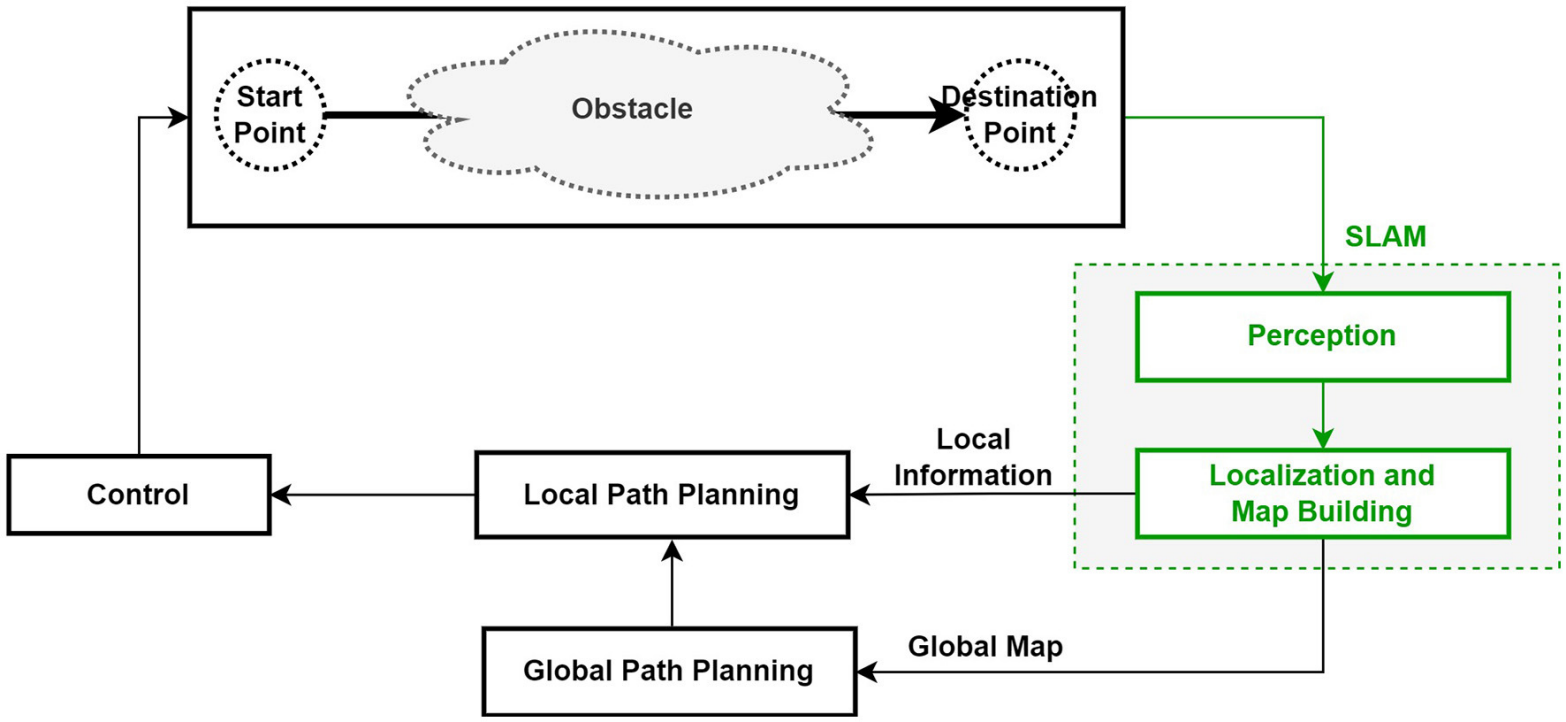
Structure of mobile robot path planning using SLAM (Zhu and Zhang, 2021).

Figure 3 describes the change in structure when the SLAM method is added to the structure in which the mobile robot utilizes the existing global path planning and local path planning.

**Figure 4:**
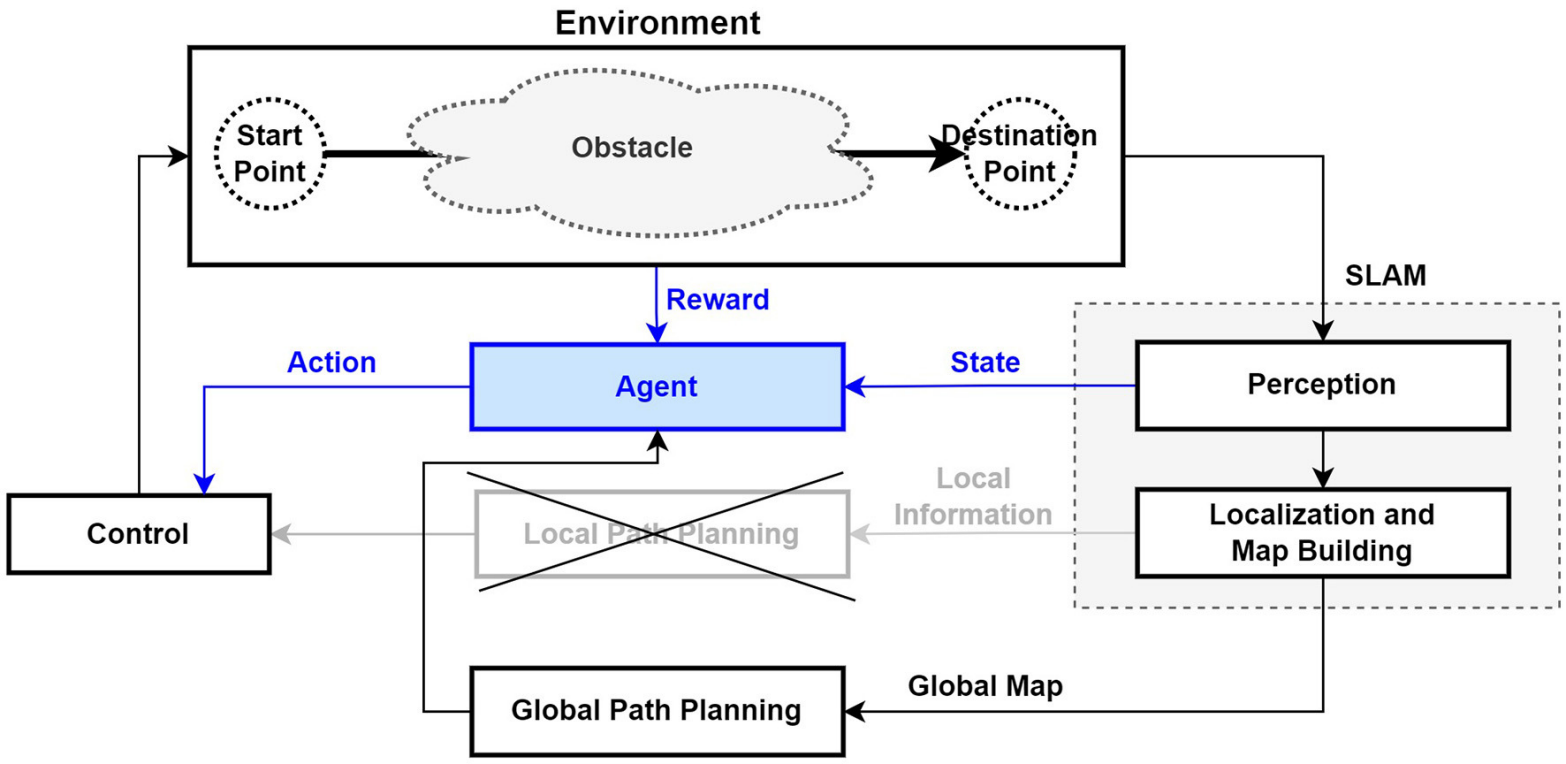
Structure of mobile robot path planning using SLAM and reinforcement learning (Zhu and Zhang, 2021).

The purpose of using a reinforcement learning algorithm for the autonomous driving of a mobile robot is to identify an optimal policy to efficiently reach a target location while interacting with the environment (Lee and Jeong, 2021). This method observes sensor information in the actual workplace environment, learns to maximize the reward value for the task, and performs autonomous driving based on this obtained sensor information and approximated information (Zhu and Zhang, 2021). Specifically, reinforcement learning involves learning through trial and error; therefore, during the learning process, the robot collides with fixed or moving obstacles and learns how to avoid them. This experimental environment also has the advantage of allowing for checking in advance through simulation testing before the actual application and operation of a mobile robot. Figure 4 shows the structure of the AMR system based on sensors and reinforcement learning as well as the interaction between the reinforcement learning agent and the environment. Instead of the existing local path planning module, the reinforcement learning agent learns to move to the target position while avoiding obstacles (Zhu and Zhang, 2021). However, in the case of a structurally very complex environment, the agent may not be able to properly learn, so it may be necessary to use global path planning to divide the entire movement path into stopovers and then set up path planning (Zhu and Zhang, 2021).

### 2.6. Reinforcement learning algorithm for path planning

In this paper, we use reinforcement learning technology for path planning. Although previous studies have established path planning for grid-type warehouse environments (Lee and Jeong, 2021; Lee et al., 2022), the goal of this paper is to create path planning for autonomous driving. In a grid-type environment, grid coordinate values have been used as state information, and discrete-type of data such as up, down, right, and left can be used as action information (Lee and Jeong, 2021; Lee et al., 2022). However, in an autonomous driving environment without grid coordinates, sensor data can be used instead of grid information as state information, while continuous-type data, such as velocity or angle, can be used for action information (Zhao et al., 2021; Zhu and Zhang, 2021).

Among various reinforcement learning algorithms, it was confirmed that the Policy-based type can handle both the Stochastic Policy and the Deterministic Policy to support continuous action space problem handling (OpenAI Spinning Up, 2018; Sutton and Barto, 2018). Specifically, it was decided to use the Deep Deterministic Policy Gradient (DDPG) and Soft Actor–Critic (SAC) algorithms in the experiment, because these algorithms have the characteristics of the Policy Gradient series and the Q-Learning series, and they are suitable for the continuous action space problem. The DDPG algorithm combines the advantages of the Deep Q-Network (DQN) algorithm and the concept of the Deterministic Policy Gradient (DPG) algorithm (Lillicrap et al., 2016; Wen et al., 2019).

**Figure 5:**
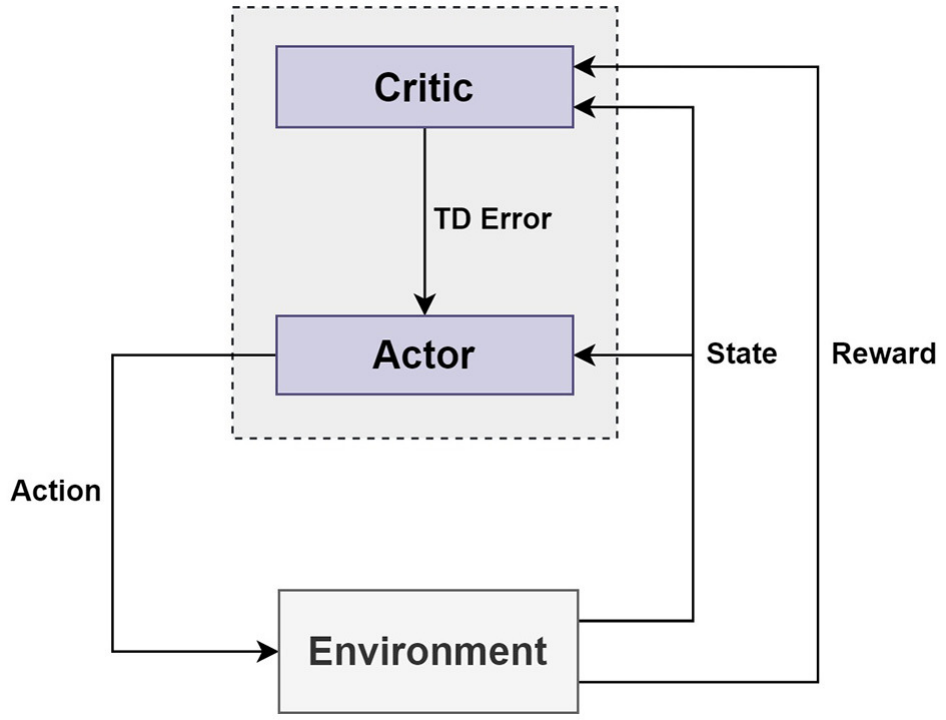
Structure of the actor–critic.

DDPG and SAC algorithms are the same types of algorithms, and they have the following common characteristics:

The first is model-free type reinforcement learning, which is an algorithm in which an agent searches to find a policy from the absence of environmental information at the beginning, then gradually learns and finds a policy through trial and error (Silver, 2015; Lillicrap et al., 2016; Sutton and Barto, 2018). The second is an off-policy algorithm. There are two types of policies for the off-policy method: the Target policy and the Behavior policy. The Target policy learns based on information such as each state and action to find an optimal policy, while the Behavior policy selects an action. If the Target and Behavior policies are different and multiple policies exist, then this is the off-policy method (Silver, 2015; Lillicrap et al., 2016; OpenAI Spinning Up, 2018; Sutton and Barto, 2018). However, if the Target and Behavior policies are the same, this means it can be the on-policy method. The third common characteristic is the Actor–Critic. The Actor–Critic algorithm is composed of two networks: an Actor network and a Critic network. The Actor network is a Policy network, while the Critic network is a Value network (Mnih et al., 2016; Sutton and Barto, 2018). The Actor-Critic algorithm has two agents, which are called Actor and Critic. The Actor and the Critic separately manage parameters; the Actor updates the policy parameters in the direction suggested by the Critic, while the Critic updates the parameters of the action–value function (Mnih et al., 2016; Sutton and Barto, 2018). The Actor decides the action given to the state, and the Critic evaluates the value of the state (Mnih et al., 2016; Sutton and Barto, 2018). The Actor and Critic can each obtain a loss value with a loss function as well as learn to minimize the total loss value by combining the two loss values (Mnih et al., 2016; Sutton and Barto, 2018). Figure 5 schematically depicts how the main elements of Actor–Critic interact.

#### 2.6.1. Deep deterministic policy gradient

The DDPG algorithm is a model-free reinforcement learning algorithm that is based on off-policy and operates in an Actor–Critic manner. It is also based on the Deterministic Policy Gradient, and it can only be used in continuous action space. The DDPG is an algorithm created by complementing the weaknesses of the Deep Q-Network (DQN) algorithm and Deterministic Policy Gradient (DPG) algorithms and taking advantage of their strengths. The DDPG algorithm is basically Actor–Critic-based and consists of an Actor network μ and a Critic network *Q*. To calculate the target value, the Target Actor network μ′ and the Target Critic network *Q*′ are also composed (Lillicrap et al., 2016; Wen et al., 2019). The Actor estimates an appropriate action from the current state, while the Critic evaluates the value of the action determined by the Actor. This is randomly sampled from the information stored while learning and trained through a deep neural network (Mnih et al., 2016; Wen et al., 2019).

As briefly explained in the Actor–Critic structure above, the DDPG algorithm learns to minimize the total loss value, and Equations 6, 7 explain how the loss value can be calculated (Lillicrap et al., 2016; Wen et al., 2019). In the formula, *s* is the state, *a* is the action, *r* is the reward value, γ is the learning rate, θ is the weight constituting the neural network, and *N* is the number of mini-batch samples. Moreover, *y*_*i*_ is the target Q-value and $Q\left(s_{i},a_{i}|\theta^{Q}\right)$ is the Estimated Q-value. The *L* is the loss value and the Mean Squared Error (MSE) value.


(6) $$y_{i}\;=\;r_{i}+\gamma Q\prime (s_{i+1},\mu \prime \left(s_{i+1}|\theta \prime^{\mu \prime }\right)|\theta^{Q\prime })$$

(7) $$L\;=\;\frac{1}{N}\sum_{i}^{}{\left(y_{i}-Q\left(s_{i},a_{i}|\theta^{Q}\right)\right)}^{2}$$

The Actor network predicts the transformation of the parameter values using the sampled policy gradient method, and it is described by Equation 8 (Lillicrap et al., 2016).


(8) $${\;{\;\nabla_{\theta^{\mu }}J\approx \frac{1}{N}\sum_{i}^{}\nabla_{a}Q\left(s,a|\theta^{Q}\right)|}_{s=s_{i},a=\mu \left(s_{i}\right)}\nabla_{\theta^{\mu }}\mu \left(s|\theta^{\mu }\right)|}_{s_{i}}$$

The DDPG algorithm has the following main features:

- Replay Buffer: The Replay buffer is denoted as R with a fixed size, and while the Agent searches for a path, it stores state, action, reward, and next state information in the form of a tuple in the Replay Buffer (Lillicrap et al., 2016; Wen et al., 2019). The Actor and the Critic learn by extracting a random mini-batch from the Replay Buffer (Lillicrap et al., 2016; Wen et al., 2019).
- Soft Target Update: In the case of DQN, there are cases in which learning is unstable because the updated Q-Network parameter value is used when calculating the Target value. To address this disadvantage, it is necessary to use a Target network that copies the Actor/Critic and a Soft Target Update method (Lillicrap et al., 2016). The copied Target network is used to calculate the Target value, and the update speed of the Target network can be adjusted by utilizing the weight value and parameter τ (Lillicrap et al., 2016). If the parameter τ is set to a small value, the copied target networks can be updated slowly, which can improve the problem of unstable learning. Equations 9, 10 are the equations that are used to update the Target network (Lillicrap et al., 2016; Wen et al., 2019). The weight of the Actor network is θ^μ^, the weight of the Critic network is θ^*Q*^, the weight of the Actor Target network is θ^μ^′, the weight of the Critic Target network is θ^*Q*^′, and τ is the approximate coefficient, which should always be < 1.

(9) $$\theta^{Q^{'}}\leftarrow \tau \theta^{Q}\;+\;(1-\tau )\theta^{Q^{'}}$$

(10) $$\theta^{\mu^{'}}\leftarrow \tau \theta^{\mu }\;+\;(1-\tau )\theta^{\mu^{'}}$$

- Batch Normalization: When the components of observation contain different physical units, this hinders network learning. To improve these constraints, batch normalization which normalizes the input and output of the layer as well as the Actor and Critic layers can be performed (Lillicrap et al., 2016).
- Noise Process: Exploration makes a new attempt at learning. In DDPG, noise is added to the Actor policy for continuous exploration, as is explained in Equation 11 (Lillicrap et al., 2016).

(11) $$a_{t}=\mu \left(s_{t}|\theta^{\mu }\right)+N_{t}$$

#### 2.6.2. Soft actor-critic

The SAC algorithm is a model-free reinforcement learning algorithm that is based on off-policy and consists of an Actor–Critic method. It is also based on Policy Gradient and can only be used in continuous action space. Existing model-free deep reinforcement learning algorithms require a new sample for each step, thus necessitating an exponentially large number of samples, ultimately resulting in low efficiency, sensitivity to changes in hyperparameters, and unstable convergence (Haarnoja et al., 2018a,b).

The SAC algorithm has the following features added to improve these disadvantages:

- Maximum Entropy Reinforcement Learning: The goal of conventional reinforcement learning is to find a policy that maximizes the expected value of the cumulative reward value. Maximum Entropy Reinforcement Learning adds the entropy of the policy for the reward policy to the existing objective function (Haarnoja et al., 2018a,b). In this case, the expected reward and entropy of the policy should be maximized, as described in Equation 12 (Haarnoja et al., 2018b).

(12) $$\pi^{*}\;=\;arg\;\underset{\pi }{\max}\sum_{t}E_{\left(s_{t},a_{t}\right)\sim \rho_{\pi }}\left[r\left(s_{t},a_{t}\right)+\alpha ℋ\left(\pi \left(\cdot |s_{t}\right)\right)\right]$$

Here, $H\left(\pi \left(\cdot |{\text{s}}^{t}\right)\right)$ represents the probability distribution entropy of an action in state *s*_*t*_ when policy π is followed. In general, to maximize the expected reward, the policy tends to be deterministic. Therefore, the randomness of the policy can be controlled with a temperature parameter, termed parameter α (Haarnoja et al., 2018a,b). Equation 13 shows the modified objective function. In the current policy π, the distribution of the state–action pair information follows ρ_π_, while γ is the discount factor (Haarnoja et al., 2018b).


(13) $$J(\pi )=\sum_{t=0}^{\infty }E_{\left(s_{t},a_{t}\right)\sim \rho_{\pi }}\left[\sum_{l=t}^{\infty }\gamma^{l-t}E_{s_{l}\sim p,a_{l}\sim \pi }\left[r\left(s_{t},a_{t}\right)+\alpha ℋ\left(\pi \left(\cdot |s_{t}\right)\right)|s_{t},a_{t}\right]\right]$$

Applying maximum entropy to the reinforcement learning goal in this way has the following advantages: First, it leads to more rapid abandonment of less probable actions than when maximizing the expected reward. On the other hand, if there is a possibility, the probability of taking action increases. Second, multiple optimal policies can be found instead of just one optimal policy.

- Soft Policy Iteration: Policy iteration is a method of learning by repeating the convergence of policy by sequentially performing policy evaluation and improvement (Haarnoja et al., 2018a,b) If the action space is finite, the soft Q value can be obtained by calculating the value of following the policy π that maximizes the maximum entropy objective in the policy evaluation, and the policy improvement updates the policy proportionally to the exponential of the soft Q function to obtain a better policy (Haarnoja et al., 2018a,b). However, if the action space is continuous, the above two methods require too much computation to converge (Haarnoja et al., 2018a).
- Soft Actor-Critic: Soft policy iteration involves performing policy evaluation and policy improvement, but because of the need for intensive computation, convergence takes a long time. To solve this problem, soft Q-function and soft policy function are approximated and optimized through deep neural networks instead of soft policy iteration (Haarnoja et al., 2018a,b). The soft Q-function is trained to minimize the soft Bellman residual and its loss function is in Equation 14 (Haarnoja et al., 2018b). The $\bar{\theta }$ is the Exponential Moving Average (EMA) value of θ obtained in the previous step and is used for stable learning (Haarnoja et al., 2018b).

(14) $$J_{Q}(\theta )=E_{\left(s_{t},a_{t}\right)\sim D}\left[\frac{1}{2}{\left(Q_{\theta }\left(s_{t},a_{t}\right)-\left(r\left(s_{t},a_{t}\right)+\gamma E_{s_{t+1}\sim p}\left[V_{\bar{\theta }}\left(s_{t+1}\right)\right]\right)\right)}^{2}\right]$$

The calculation of the above formula with Stochastic Gradient Descent (SGD) is equivalent to that shown in Equation 15 (Haarnoja et al., 2018b). The soft policy function is learned in the direction of reducing KL divergence. The loss function is shown in Equation 16 below (Haarnoja et al., 2018b).


(15) $$\begin{array}{l} \;{\hat{\nabla }}_{\theta }J_{Q}(\theta )\\ =\nabla_{\theta }Q_{\theta }\left(a_{t},s_{t}\right)(Q_{\theta }\left(s_{t},a_{t}\right)-\left(r\left(s_{t},a_{t}\right)+\gamma \left(Q_{\bar{\theta }}\left(s_{t+1},a_{t+1}\right)-\alpha log\left(\pi_{\phi }\left(a_{t+1}|s_{t+1}\right)\right)\right)\right) \end{array}$$

(16) $$J_{\pi }(\phi )=E_{s_{t}\sim D}\left[E_{a_{t}\sim \pi_{\phi }}\left[\alpha log\left(\pi_{\phi }\left(a_{t}|s_{t}\right)\right)-Q_{\theta }\left(s_{t},s_{t}\right)\right]\right]$$

Since a Q-function is used for the target part, π is also parameterized using the same neural network transform as Q and described in Equation 17. ϵ_*t*_ is the input noise vector (Haarnoja et al., 2018b). After that, the loss function J_π_ is changed to Equation 18, and the gradient of the policy objective is approximated as in Equation 19 (Haarnoja et al., 2018b).


(17) $$a_{t}=f_{\phi }\left(\epsilon_{t};s_{t}\right)$$

(18) $$J_{\pi }(\phi )=E_{s_{t}\sim D,\epsilon_{t}\sim N}\left[\alpha log\pi_{\phi }\left(f_{\phi }\left(\epsilon_{t};s_{t}\right)|s_{t}\right)-Q_{\theta }\left(s_{t},f_{\phi }\left(\epsilon_{t};s_{t}\right)\right)\right]$$

(19) $$\begin{array}{l} \;{\hat{\nabla }}_{\phi }J_{\pi }(\phi )\\ =\nabla_{\phi }\alpha log\left(\pi_{\phi }\left(a_{t}|s_{t}\right)\right)+\left(\nabla_{a_{t}}\alpha log\left(\pi_{\phi }\left(a_{t}|s_{t}\right)\right)-\nabla_{a_{t}}Q\left(s_{t},a_{t}\right)\right)\nabla_{\phi }f_{\phi }\left(\epsilon_{t};s_{t}\right) \end{array}$$

## 3. Methods

### 3.1. System architecture

As explained earlier, the HW part should be able to detect the external environment by embedding a LiDAR sensor, and the path planning of the mobile robot should be established through trial and error by utilizing the detected external environment using reinforcement learning. For this purpose, the structure of the Autonomous Mobile Robot Framework is mainly composed of two layers: HW controller, which directly controls the robot, and SW controller, which is based on agents. The HW part includes a LiDAR sensor, and the SW controller part also includes deep reinforcement learning part to enable path-planning learning.

**Figure 6:**
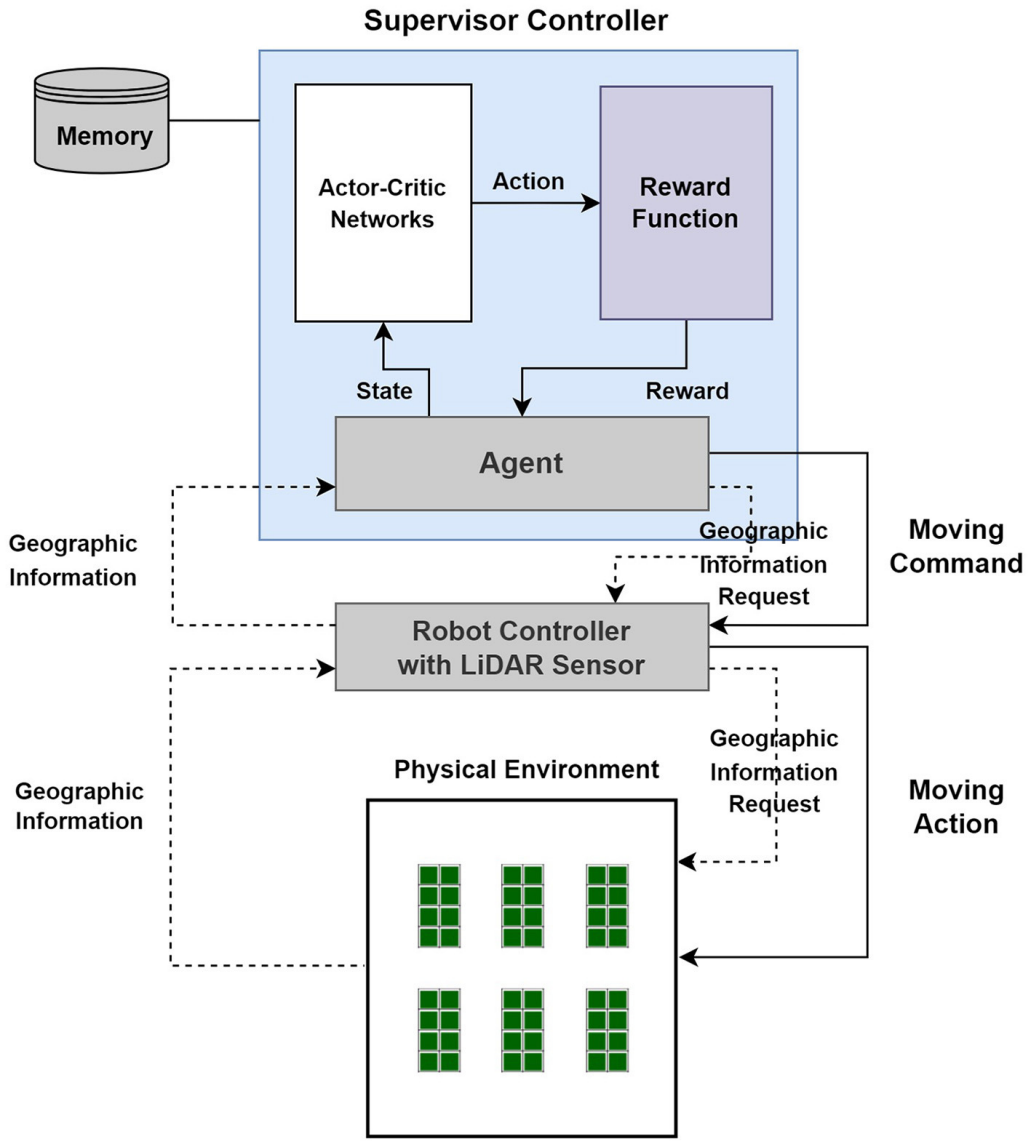
Autonomous mobile robot framework in a warehouse (Lee and Jeong, 2021; Lee et al., 2022).

Figure 6 depicts the structure including the software/hardware architecture and the warehouse environment in which the autonomous mobile robot is operated. The robot controller that controls the actual hardware is the part that physically controls the movement of the mobile robot, and the part that collects local environment information using LiDAR sensors is considered. For reference, the proposed architecture only considers local path planning and does not consider global path planning.

In the structure of the Autonomous Mobile Robot Framework in Figure 6, it will work as follows:

- The agent requests geographic information from the robot controller, which is delivered to the LiDAR sensor attached to the robot to check the external environment information of the site. The robot controller delivers the geographic information, which is the external environment information confirmed by the LiDAR sensor, to the requesting agent.
- The agent inputs some of the geographic information, which is the external environment information confirmed by the LiDAR sensor, into the Actor-Critic network to receive an action value and inputs this value into the reward function to receive a reward value and next state information.
- Based on the received reward value and the following state information, the agent sends a moving command to the robot controller, and the mobile robot moves.

### 3.2. State function and network structure

State information *s*_*t*_ is defined as expressed in Formula 20 as a translation function formula that takes distance data *x*_*t*_ and relative target coordinate information *p*_*t*_ about the target as input values (Lei et al., 2017; Grando et al., 2021).


(20) $$\begin{array}{l} s_{t}\;=\;f\left(x_{t},p_{t}\right) \end{array}$$

The state information defined in this way can be input to the deep neural network and the velocity and angular velocity values can be approximated with action values. The DDPG and SAC algorithms which the reinforcement learning algorithms used in this paper define the network structure to use the value obtained as the output information as the action value by inputting state information as the input parameter of the policy network, which is an Actor network (Lei et al., 2017; Grando et al., 2021; Han et al., 2021).

**Figure 7:**
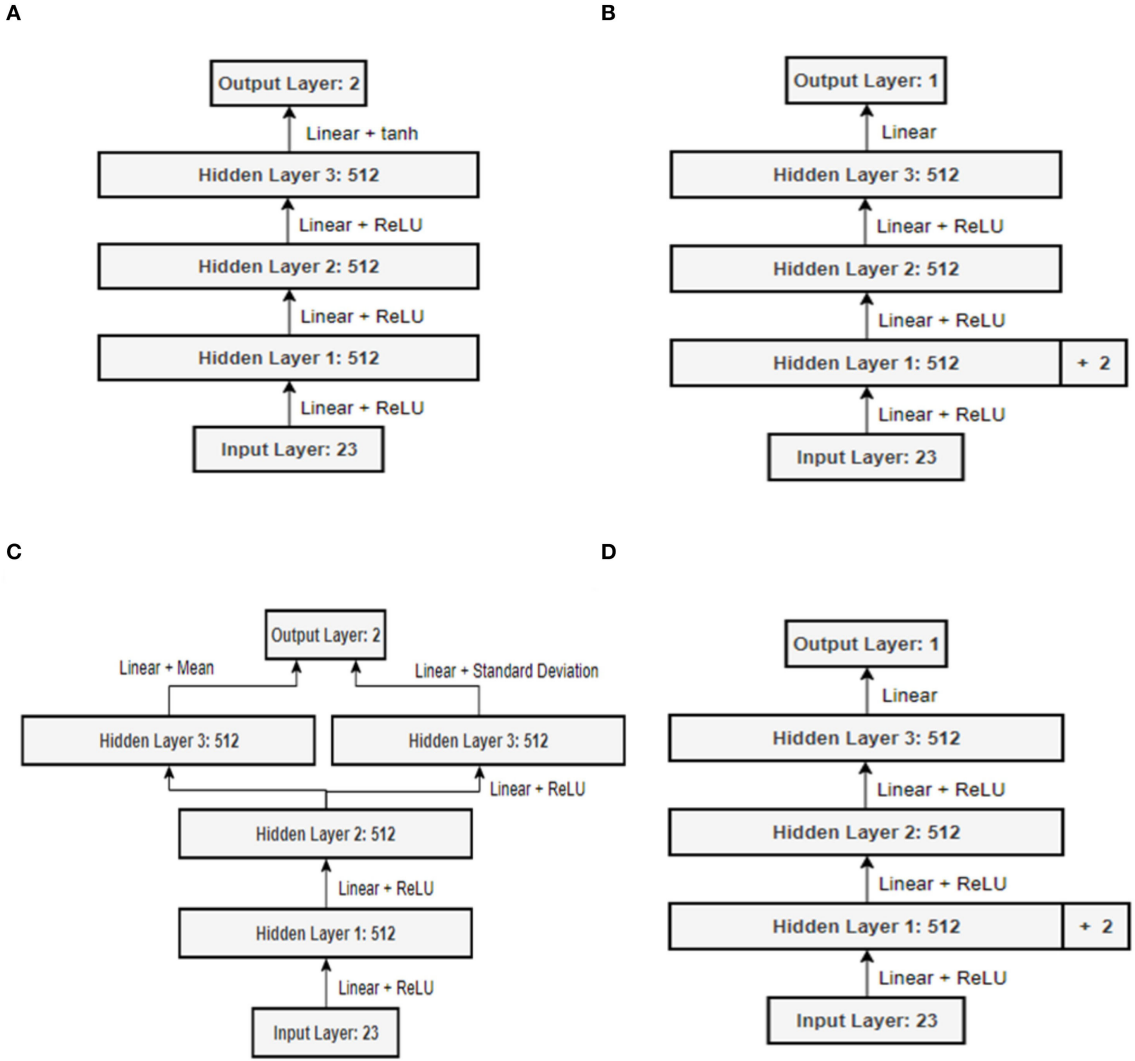
Neural network structure: **(A)** Policy network of DDPG; **(B)** Q-network of DDPG; **(C)** Policy network of SAC; **(D)** Q-Network of SAC.

In the case of the Q-Network structure, state information, action information, and the output value obtained through the Policy network were used as the input parameters (Zhang et al., 2019). In relation to this, by adding two nodes at the end of the first hidden layer, it was designed to concatenate and input action information, which is Policy network output information (Zhang et al., 2019). Figure 7 explains the Actor and Critic network structures of the DDPG and SAC algorithms used for the experiments in this paper. After three fully connected neural network layers with 512 nodes, the input values are converted into velocity and angular velocity information, and the hyperbolic tangent function tanh is used as the activation function to limit the range of angular velocity in the range −1 to 1 (Zhang et al., 2019; Han et al., 2021; Liu et al., 2022).

### 3.3. Reward function

The mobile robot continues to move the step of the minimum movement unit and arrives at the final target position. After the mobile robot starts to move at the beginning, it receives the step reward value for each step movement and accordingly determines the direction and speed of the next movement. When the mobile robot reaches the final target position, the movement of the episode ends, and a comprehensive reward value for the path trajectory that has been traveled from the beginning to the arrival is given, and this is called the final reward value. It is a comprehensive reward value that is given when the final arrival or failure to the destination is within a single episode.

In general, if it arrives at the target position normally, the final reward value is given a positive reward value; meanwhile, in the case of failure or failure to reach the target position, such as in a collision, a negative reward value or a relatively low reward value is given as a penalty (OpenAI Spinning Up, 2018; Wen et al., 2019).

For the symbols used in the following formulas, we have added a short description as follows: *r*_*final*_*reward*_( ) is the reward function to calculate the final reward value, *r*_*step*_*reward*_( ) is the reward function to calculate the step reward value, s_*t*_ is the 21 channel distance values of mobile robot, a_*t*_ is the action values obtained based on 21 channel distance values of mobile robot, *d*_*t*_ is the distance between the location of the mobile robot and the target position, *o*_*t*_ is the arctan value for the angle between the target position and the moved position, *c*_*d*_ is the threshold distance value to determine whether mobile robot is arrived, *c*_*o*_ is the threshold distance value to determine whether mobile robot is collide, *min*_*x*_ is the smallest distance value of 21 channels, *r*_*arrive*_ is the reward value when mobile robot arrives to the target position, and *r*_*collide*_ is the reward value when mobile robot collide.

As shown in Equation 21, if *d*_*t*_ is smaller than *c*_*d*_, then it is determined that the robot has arrived at the target position, and a positive reward value *r*_*arrive*_ is given (OpenAI Spinning Up, 2018; Wen et al., 2019). A collision is considered to be detected when *min*_*x*_ is smaller than *c*_*o*_, and a negative reward value *r*_*collide*_ is given (Lei et al., 2017; Sharma, 2020; Grando et al., 2021).


(21) $$r_{final\_reward}\left(s_{t},a_{t}\right)=\{\begin{array}{cccc} & r_{\text{arrive }} & & \text{ if }d_{t}<c_{d}\\ & r_{\text{collide }} & & \text{ if }\min_{x}<c_{o} \end{array}$$

The step reward value is a reward value that is given when the mobile robot continuously moves in a minimum unit to reach the final target position. The most basic step reward value can be defined as a composite value of *d*_*t*_ and *o*_*t*_. At this time, if importance weight values can be used for the *d*_*t*_ and *o*_*t*_ values, the step reward value can be controlled in a more flexible manner. *w1* and *w2* are weight values that can be defined in the range 0 to 1.

In this paper, to increase the additional learning efficiency for reinforcement learning, we proposed “Velocity Range-based Evaluation Method” that reflects the value evaluated as the action value in the basic step reward for the distance and angle to the target position. Among the action values, the velocity value is divided into several sections based on the percentage in the range −100 to 100%, while the evaluation score value is differentially defined for each divided section. Equation 22 defines a step reward that reflects the evaluation score value *e*_*t*_, and its value is min −1 and max 1.


(22) $$r_{step\_reward}\left(s_{t},a_{t}\right)=\;\left(w1\times d_{t}+w2\times o_{t}\right)+\;e_{t}$$

The guide is a detailed description how to define velocity ranges and its evaluation scores for proposed “Velocity Range-based Evaluation Method” below.

**Table:** 

Guide: Define velocity ranges and evaluation scores
*1. Divide −100 to 100% into several ranges for actual velocity values*. *It is assumed that the number of ranges is n, and the divided ranges* *stand for v_1_ to v_*n*_*. *v_1_ starts from −100% and v_*n*_ ends at 100%*. *(v_1_ < v_2_ < … < v_*n*_)* *2. Define evaluation score values*. *It is assumed that the defined evaluation score values stands for* *e_1_, e_2_, ….., e_*n*_*. *(e_1_ < e_2_ < … < e_*n*_)* *3. Match one of the evaluation score values for each divided percentage velocity* *range*. *A higher evaluation score value is assigned to a relatively high percentage* *velocity range*. *(v_1_ to e_1_, v_2_ to e_2_, … , v_*n*_ to e_*n*_)*

The following algorithm is the training part of reinforcement learning. (c), (d), and (e) describe how to use “Velocity Range-based Evaluation Method”.

**Algorithm 1:**
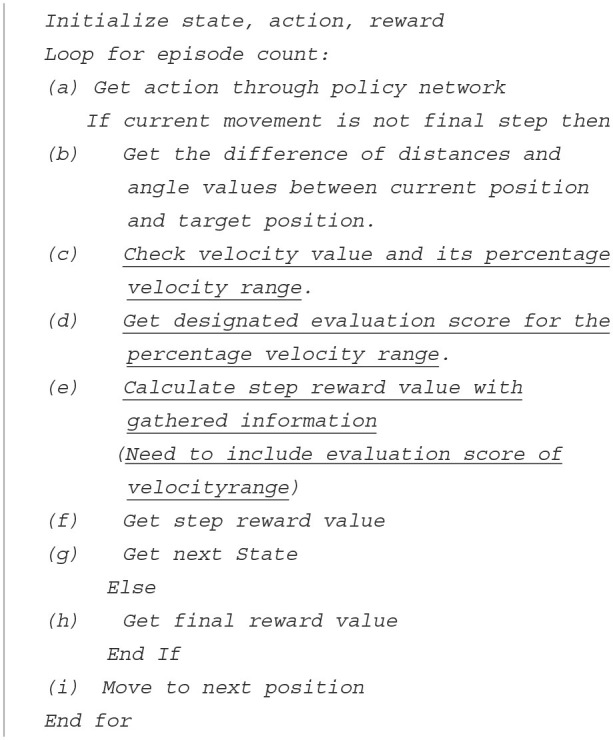
Training with velocity range-based evaluation method.

### 3.4. Experiment environment

**Figure 8:**
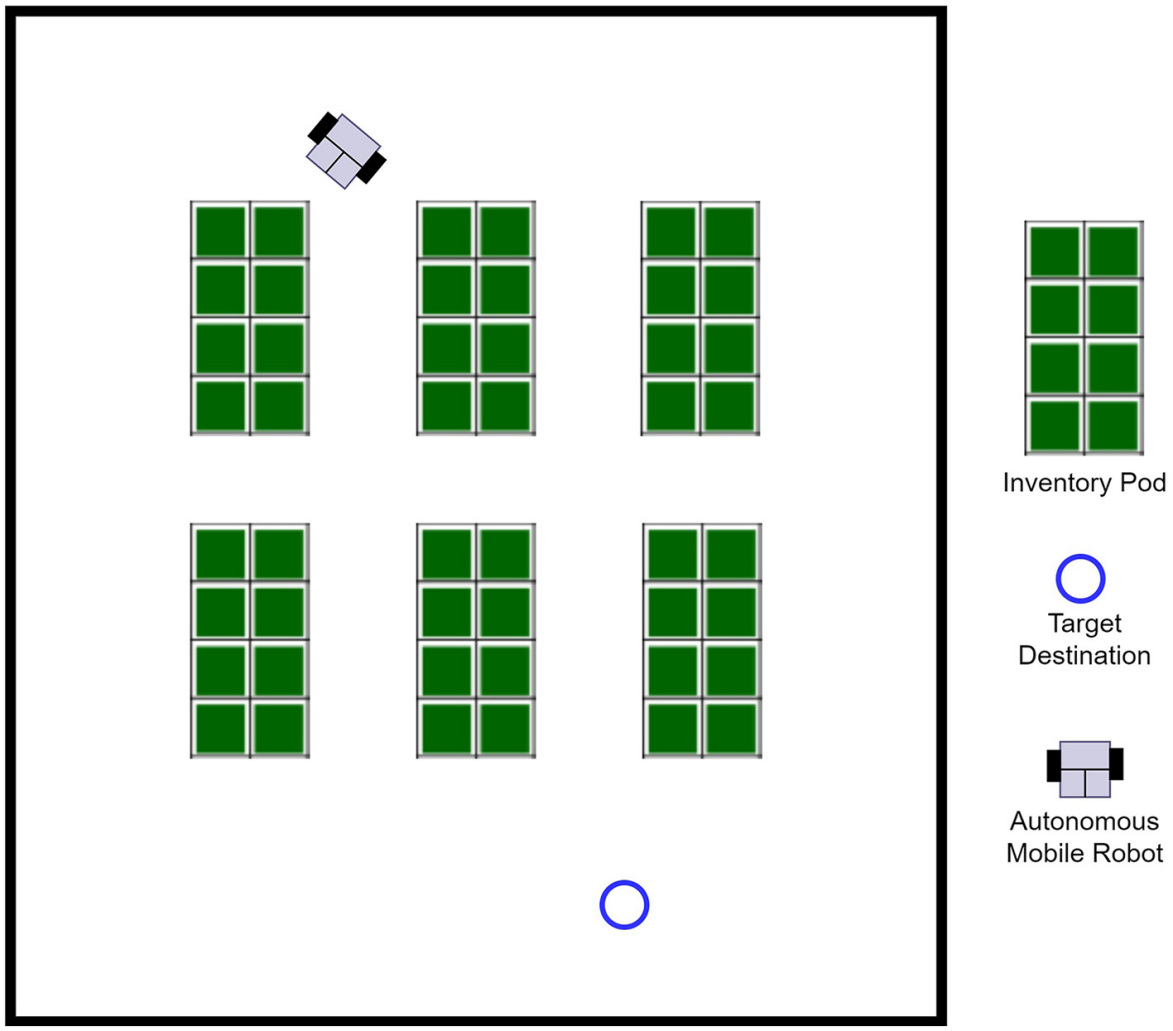
Experimental environment.

The experimental environment used in this paper was configured based on a warehouse environment. The warehouse layout was configured as a simulation environment for a small-scale work environment with six inventory pods in a simple form. An open source simulation was used to build the experimental environment, and Figure 8 shows the layout of the environment.

Moreover, the LiDAR sensor simulation for the experiment assumed the use of a LiDAR sensor composed of 21 laser channels between −120 and 120 degrees (Lei et al., 2017; Kim et al., 2021).

### 3.5. Experiment method

In this experiment, the starting position of the mobile robot's movement and the target position to which it will arrive are continuously changed randomly for each episode, and a single mobile robot avoids the inventory pod, and proceeds to find the optimal autonomous driving path to the target location.

The experiments are performed in two cases below:

- Experiment #1: Experiments are performed on the DDPG and SAC algorithms without applying the “Velocity Range-Based Evaluation Method”. LiDAR sensor and lowering the performance of the part.
- Experiment #2: The experiment applies the “Velocity Range-Based Evaluation Method” to experiment with DDPG and SAC algorithms.

For our experiments, we defined the following parameter values. As a default Final Reward Value, we defined 20 for reaching the target position and −2 for collision. Moreover, in relation to the proposed technique “Velocity Range-based Evaluation Method” in the step reward, the evaluation score value in Table 1 was applied and used in the experiment. Table 2 shows the Hyper Parameter values used in the DDPG and SAC algorithms in the experiment detailed in this paper.

**Table 1:** Evaluation score of percentage velocity range.

**Velocity range**	**Evaluation score**
−100% ≤ *v_1_* < −70%	−0.3
−70% ≤ *v_2_* < −30%	−0.1
−30% ≤ *v_3_* < 0%	−0.05
0% ≤ *v_4_* < 30%	0
30% ≤ *v_5_* < 70%	0.05
70% ≤ *v_6_* < 100%	0.1

**Table 2:** Parameter values for experiment.

**Parameter**	**DDPG**	**SAC**
Learning rate (Actor)	0.00005	0.00005
Learning rate (Critic)	0.00005	0.00005
τ Value	0.01	0.01
Memory size	100,000	100,000
Batch size	64	64
Max epoch	5,000	5,000
α value	N/A	0.1

### 3.6. Performance metrics

The experiments were performed three times with the given conditions for each algorithm. The evaluation method used to confirm the experimental results was evaluated and analyzed based on the following indicators.

- The trend of path movement count: This is the value of how many movements were required to reach the target position if a path was found in a single episode. It is an indicator for checking how quickly the path was found during learning. We provide two kinds of visualization graphs; simple plots and box plots. For the simple plot, the value was obtained by dividing every 50 episodes and calculating their average value. For the box plot, the value was obtained by dividing every 500 episodes and it shows maximum value, minimum value, quartile values, and outlier values at every interval. The results of the three experiments were independently plotted and displayed on a single graph; the results of the DDPG and SAC algorithms are displayed in separate graphs.
- The trend of final reward value: This is the reward value given when a path was found in a single episode. It is an indicator that checks the level of learning as learning progresses. The description of the visualization graph's types, the information the graphs provide, and the types of experiments the graphs show are the same as the description of the trend of path movement count above.
- The comparison of the path movement count's average: This is the overall average value of path movement counts of three times conducted experiments. We provide two kinds of visualization graphs; simple plots and box plots. For the simple plot, the average value was obtained by dividing every 50 episodes for each experiment and it shows the overall average value of three experiments. For the box plot, the value was obtained by dividing every 500 episodes and it shows the overall maximum value, minimum value, quartile values, and outlier values of three experiments at every interval. A single graph includes the following four cases: experiment #1 with DDPG, experiment #1 with SAC, experiment #2 with DDPG, and experiment #2 with SAC.
- The comparison of the final reward value's average: This is the overall average value of final reward values of three times conducted experiments. The description of the visualization graph's types, the information the graphs provide, and the types of experiments the graphs show are the same as the description of the comparison of the path movement count's average above.
- The path movement count during the learning stabilization phase: In the case of learning with the SAC algorithm, it was judged that the stabilization phase of learning was entered from about the 2,000th episode, and data from episodes 2,000 to 5,000 were checked for this item. The results of three replications of experiments were independently plotted and displayed on separate graphs. This was checked by separating and visualizing the results before and after applying the “Velocity Range-based Evaluation Method” to the experiment on the SAC algorithm.
- The comparison of the cumulative frequency for an inefficient learning path: First of all, when experimenting with the SAC algorithm, learning became stable from about the 2,000th episode, so stable data from episodes 2,000 to 5,000 were checked, excluding data in an unstable state at the beginning of learning. At this time, if the path movement count was over 150, the learning was judged as being inefficient. Therefore, the case in which the path movement count exceeds 150 is divided into nine ranges: (150–200), (200–250), (250–300), (300–350), (350–400), (400–450), (450–500), (500–550), and (550–600); the frequency for each range was checked. At this time, all three experimental data were combined and checked, and the frequency of episodes was verified by experimenting with and without applying the proposed “Velocity Range-based Evaluation Method” technique.
- The comparison of the path found rate: Three experiments were conducted with the SAC algorithm and the “Velocity Range-based Evaluation Method” applied to the SAC algorithm. Each time, the experiment was repeated for 5,000 episodes, and the percentage of successfully found paths was calculated every 50 episodes. In addition, the maximum and minimum percentages of the three experiments were visualized in a graph to check the deviation between the experiments.

## 4. Results and discussions

**Figure 9:**
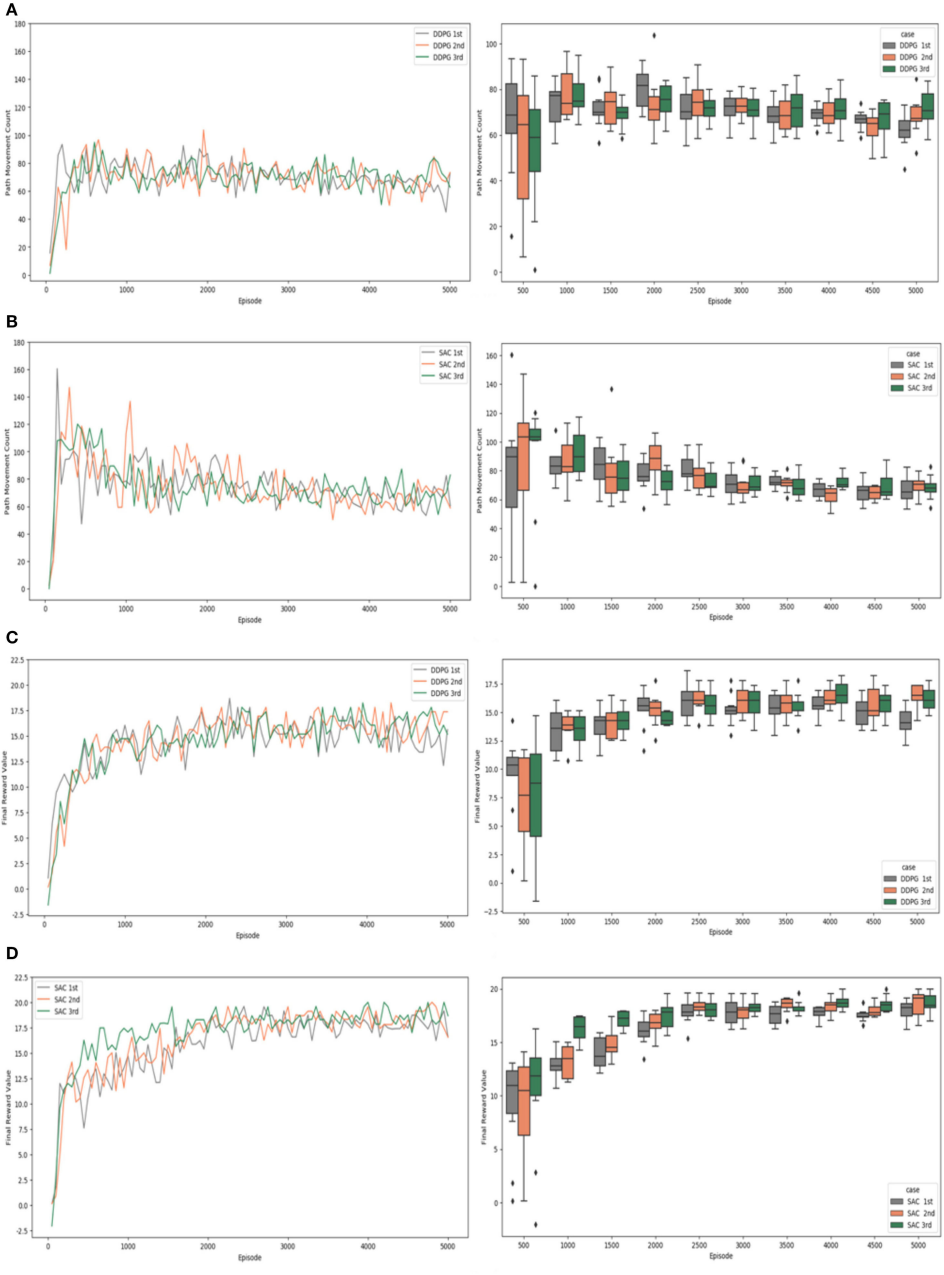
Experiment #1 result: **(A)** path movement count of DDPG; **(B)** path movement count of SAC; **(C)** final reward value of DDPG; **(D)** final reward value of SAC.

**Figure 10:**
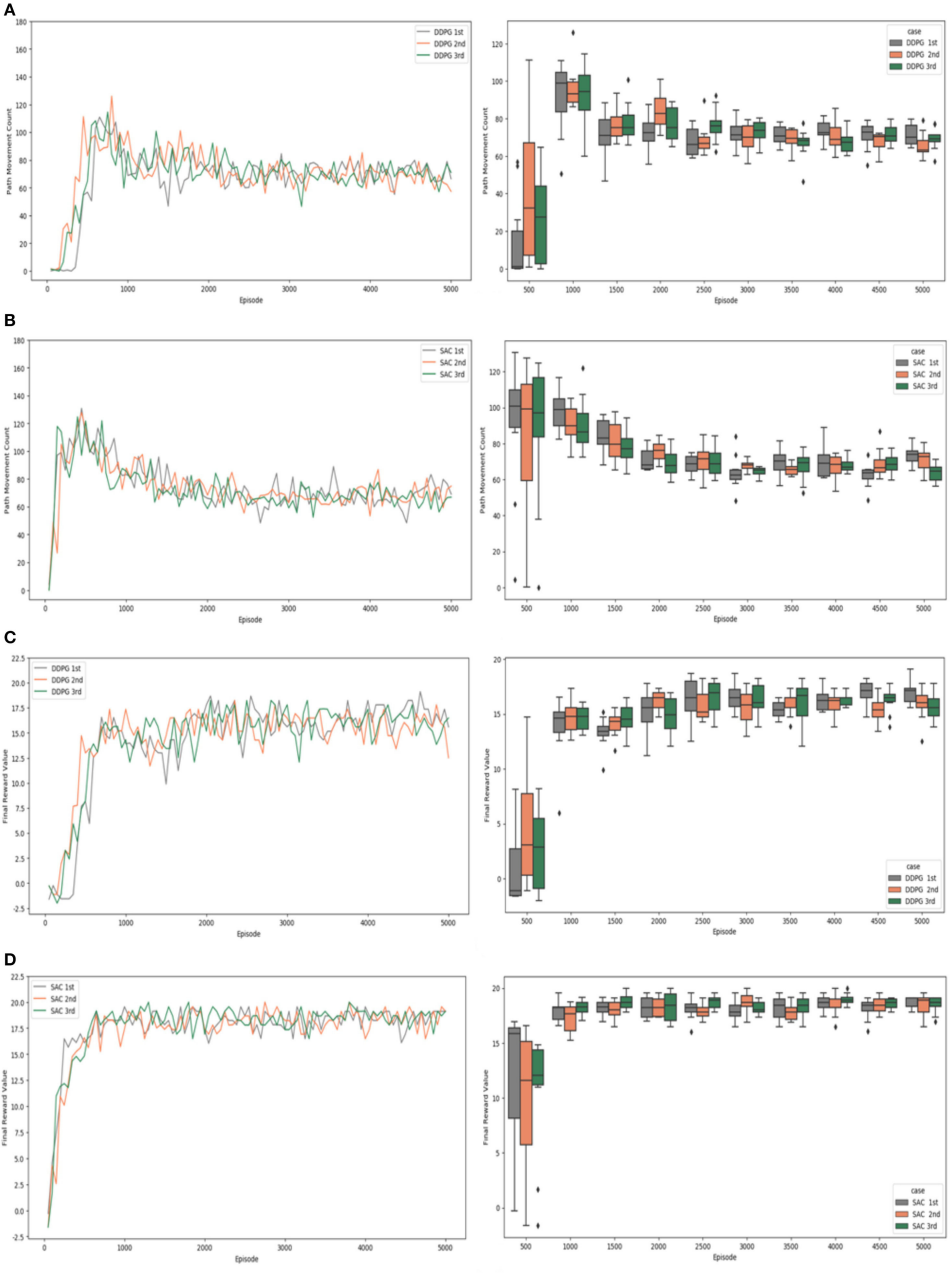
Experiment #2 result: **(A)** path movement count of DDPG; **(B)** path movement count of SAC; **(C)** final reward value of DDPG; **(D)** final reward value of SAC.

Figures 9, 10 check and visualize the path movement count and the final reward value from the experimental results by a simple plots and a box plots. The results of the experiments that were conducted are detailed in the following. Figure 9 shows the results of experiment #1, which did not apply “Velocity Range-based Evaluation Method”.

According to the path movement counts of the DDPG algorithm experiment in a simple plot graph of Figure 9A, the values fluctuate between 60 and 100 before the 1,000th episode, and the values fluctuate between 80 and 60 as learning progresses. The path movement counts of the SAC algorithm experiment in a simple plot graph of Figure 9B showed values with very large deviations as well as wide fluctuations in the early stages of learning, and the number of steps increased to 160. However, as learning progressed, the changes in the values decreased, and it was confirmed that they stably converged to values between 80 and 60 from about the 3,000th episode. In the early stage of learning, the DDPG algorithm showed a relatively small range of value fluctuations and reached the target position with a small number of steps, but the experimental results of the SAC algorithm and the DDPG algorithm showed similar performance after the 3,000th episode. In the box plots, the width of the quartiles and the difference between the minimum and maximum values were found to be slightly narrower for SAC algorithm compared to DDPG algorithm.

In Figure 9C, which shows the final reward values of the DDPG algorithm experiment in a simple plot graph, it can be seen that the reward value increases at the beginning of learning and learning proceeds, and the reward value fluctuates between 13 and 18 after the 2,000th episode and is maintained until the end. The final reward values of the SAC algorithm experiment shown in a simple plot graph of Figure 9D learned by increasing the reward value from the beginning, but the reward value fluctuated between 16.5 and 20 after the 2,000th episode and was maintained until learning ended. According to the box plot, the difference is obvious from the 2,000th episode. The width of the quartiles and the difference between the minimum and maximum values are narrower for SAC compared to DDPG. It can also be seen that the positions of the boxes become quite similar in the case of the SAC algorithm.

Figure 10 shows the results of experiment #2 which “Velocity Range-based Evaluation Method” was applied. The path movement counts of the DDPG algorithm experiment in a simple plot graph are shown in Figure 10A. The values fluctuated between 60 and 100 around the 1,000th episode, while the values fluctuated between 80 and 60 as learning progressed. The path movement counts of the SAC algorithm experiment in a simple plot graph are shown in Figure 10B. As learning progressed, the values stabilized, and it was confirmed that they stably converged to values between 80 and 60 after the 3,000th episode. In the early stage of learning, the DDPG algorithm showed a relatively small range of value fluctuations and reached the target position with a small number of steps, but after the 3,000th episode, the experimental results of the SAC algorithm and the DDPG algorithm showed similar performance. Checking the experimental results in the box plots, we can see that the width of the quartiles and the difference between the minimum and maximum values are slightly narrower for SAC compared to DDPG in experiment #1, but they are quite similar in experiment #2.

According to the DDPG algorithm experiment's final reward values shown in Figure 10C, the reward value increased at the beginning of learning and learning progressed. The reward values fluctuated and were maintained between 13 and 18 after the 2,000th episode. In the case of the SAC algorithm experiment's final reward values shown in a simple plot graph of Figure 10D, learning proceeded with the reward value rapidly increasing before the 500th episode. The reward values fluctuated and were firmly maintained between 17 and 20 after the 2,000th episode. The box plots show that the differences start to become apparent after 2,000 episodes. The width of the quartiles and the difference between the minimum and maximum values are narrower for SAC compared to DDPG. Also, in the case of the SAC algorithm, after 3,500 episodes, the locations of the boxes are clustered around the reward value of 20, and the median values are quite similar, showing a consistent data distribution.

**Figure 11:**
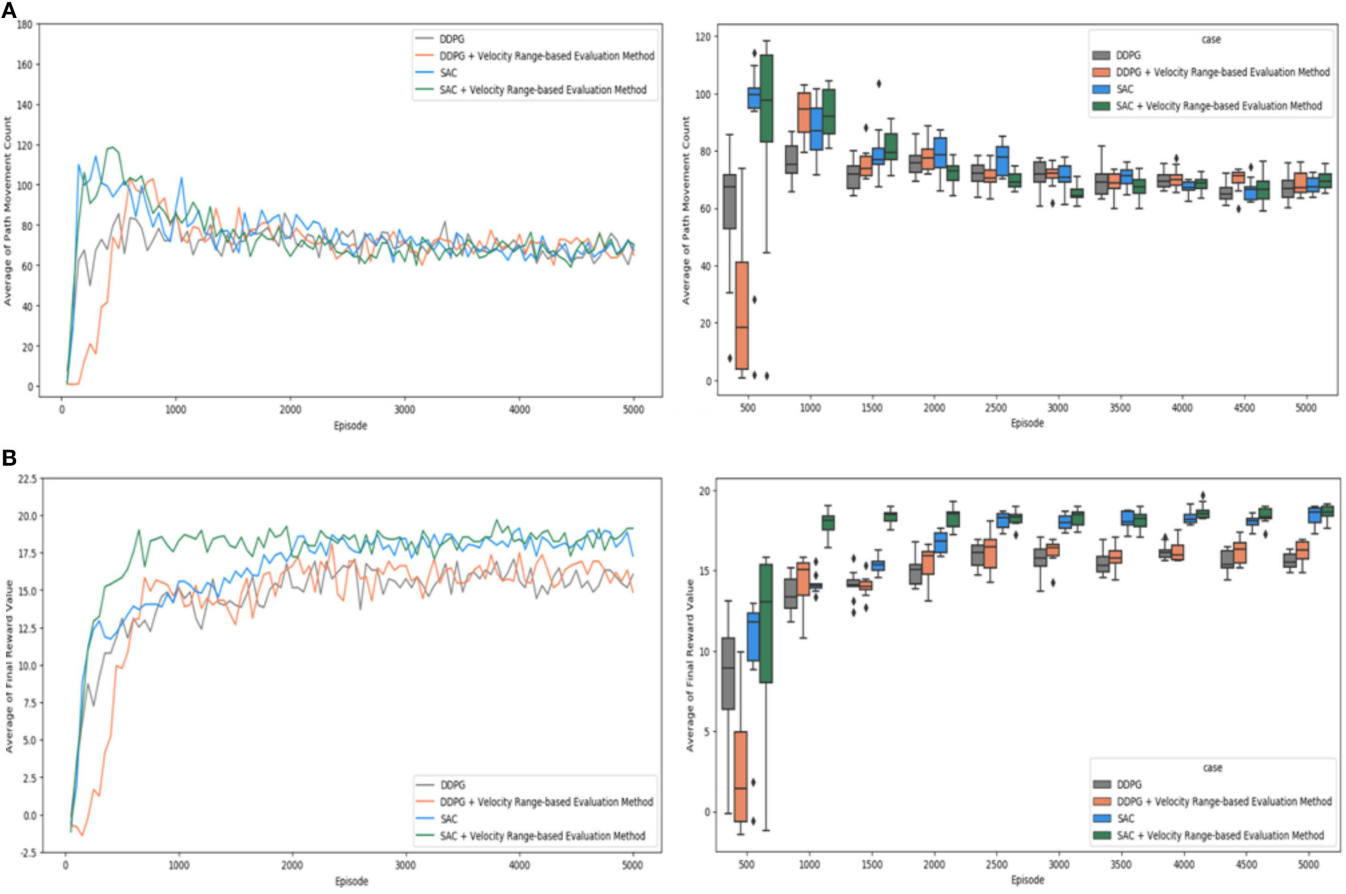
Comparison of test cases: **(A)** average of path movement count; **(B)** average of final reward value.

In the graphs shown above, the results of individual experiments can be verified, and the DDPG algorithm and the SAC algorithm can be compared in terms of the evaluation criteria. However, it is not easy to grasp and understand the meaning and implications of the overall experimental results. Therefore, as shown in Figure 11, the average values of the results of three experiments were verified for each evaluation criterion and comprehensively compared in a single graph without distinguishing between the algorithms. Figure 11 verify and visualize the path movement count and the final reward value by a simple plots and a box plots.

The averages path movement count values in a simple plot graph are shown for each experimental case in Figure 11A. Compared to the SAC algorithm, the DDPG algorithm tends to reach the target position with a relatively smaller number of steps in the initial learning. However, both the SAC algorithm and the DDPG algorithm showed similar performance after the 3,000th episode. It was confirmed that the count of path movements converged to about 60 to 80 after the 3,000th episode. In these results, there was not much difference between applying the “Velocity Range-based Evaluation Method” and not applying it. Moreover, there was no significant difference in the experimental results between the DDPG algorithm and the SAC algorithm. In the box plot graph, the differences in the size of the boxes and their values are not significant after 3,000th episode.

The average final reward values in a simple plot graph are shown for each experimental case in Figure 11B, where it can be seen that the SAC algorithm shows definitely superior performance than the DDPG algorithm. In particular, the experiment using the “Velocity Range-based Evaluation Method” shows slightly more stable and superior performance than the case in which it is not applied. When the proposed technique was applied, it was confirmed that the learning proceeded robustly from the beginning of learning and was maintained stably. In the simple plot graph, the “Velocity Range-based Evaluation Method” definitely learns quickly and shows good performance before the 2,000th episode, but it is not clear whether the improvement is valid after the 2,000th episode. In the box plot graph, we can clearly see that the SAC algorithm performs better than DDPG, but it is not easy to make sure if the experiment that applied “Velocity Range-based Evaluation Method” has improved, too. Therefore, further verification is needed to confirm the validity of the “Velocity Range-based Evaluation” Method when testing with the SAC algorithm.

Simple plot graphs and box plot graphs were provided to understand and verify the experimental results. We roughly identified which experiments were superior and which were inferior. However, it might not be clear enough for some points to check the validity with only two visualization graphs, so we performed statistical validation. To determine whether the observed results are statistically significant, we conducted statistical significance tests by paired *t*-test. The test was performed with a confidence level of 0.95 and α value of 0.05.

- Regarding the path movement count, two points need to be confirmed. The first is to verify that the experimental results of DDPG and SAC are similar after 3,000 episodes. The null hypothesis “the experimental results of DDPG and SAC are similar after 3,000 episodes” is confirmed to be true for experiment #1 and experiment #2. For experiment #1, the p one-tailed test value is 0.4632, and it confirmed that the null hypothesis is true. For experiment #2, the p one-tailed test value is 0.0638, and it confirmed that the null hypothesis is true. Therefore, the experimental results of DDPG and SAC proved not significantly different after 3,000th episode in terms of path movement count. The second is to verify that there is no significant difference between the “Velocity Range-based Evaluation Method” applied to the SAC algorithm and the SAC algorithm after 3,000th episode in terms of path movement count. Therefore, the null hypothesis “the experimental results of the ‘Velocity Range-based Evaluation Method' applied to the SAC algorithm and the SAC algorithm are similar after 3,000 episodes” is confirmed to be true. The p one-tailed test value is 0.4521, and it confirmed that the null hypothesis is true. Therefore, the experimental results of the “Velocity Range-based Evaluation Method” applied to the SAC algorithm and the SAC algorithm proved no significantly different after 3,000th episode in terms of path movement count.
- Regarding the final reward value, two points need to be confirmed. The first is to verify whether the performance of the SAC algorithm is better than the DDPG algorithm in terms of the final reward value. To check this, we verified the null hypothesis “the final reward value of the DDPG algorithm and the final reward value of the SAC algorithm are the same” and the alternative hypothesis “the final reward value of the SAC algorithm is greater than the final reward value of the DDPG algorithm”. For the result of paired *t-*test, the p one-tailed test value is 1.8753E-10, and it confirmed that the null hypothesis is false and the alternative hypothesis is true. The second is to verify whether the “Velocity Range-based Evaluation Method” performs better in terms of the final reward value. To check this, we verified the null hypothesis “the final reward value of the SAC algorithm and the final reward value of the ‘Velocity Range-based Evaluation Method' applied to the SAC algorithm are the same” and the alternative hypothesis “the final reward value of the ‘Velocity Range-Based Evaluation Method' applied to the SAC algorithm is greater than the final reward value of the SAC algorithm.” For the result of paired *t-*test, the p one-tailed test value is 0.0023, and it confirmed that the null hypothesis is false and the alternative hypothesis is true. Consequently, it is proved that the SAC algorithm performs better than the DDPG algorithm, and the “Velocity Range-based Evaluation Method” applied to the SAC algorithm performs better than the SAC algorithm only.

From the above visualization and statistical analysis, it is clear that the SAC algorithm outperforms the DDPG algorithm in terms of the trend of the final reward value and it was confirmed that the final reward value was higher when the proposed method “Velocity Range-based Evaluation Method” was applied than when it was not applied. However, in terms of the effectiveness of the “Velocity Range-based Evaluation Method”, it would be better to check what specific problems were improved in addition to better learning, stability, and higher reward values.

If we look closely at the simulation experiment after the 2,000th episode, the mobile robot mostly finds the path quickly. However, finding the path was delayed intermittently and it takes more time to arrive at the target position sometimes. In this paper, we will name this problem “Intermittent Path Movement Count Increase Problem”. Therefore, we checked whether the proposed “Velocity Range-based Evaluation Method” is effective in improving this issue. For this, we checked the movement counts for all paths found in the experimental results in a bar graph.

**Figure 12:**
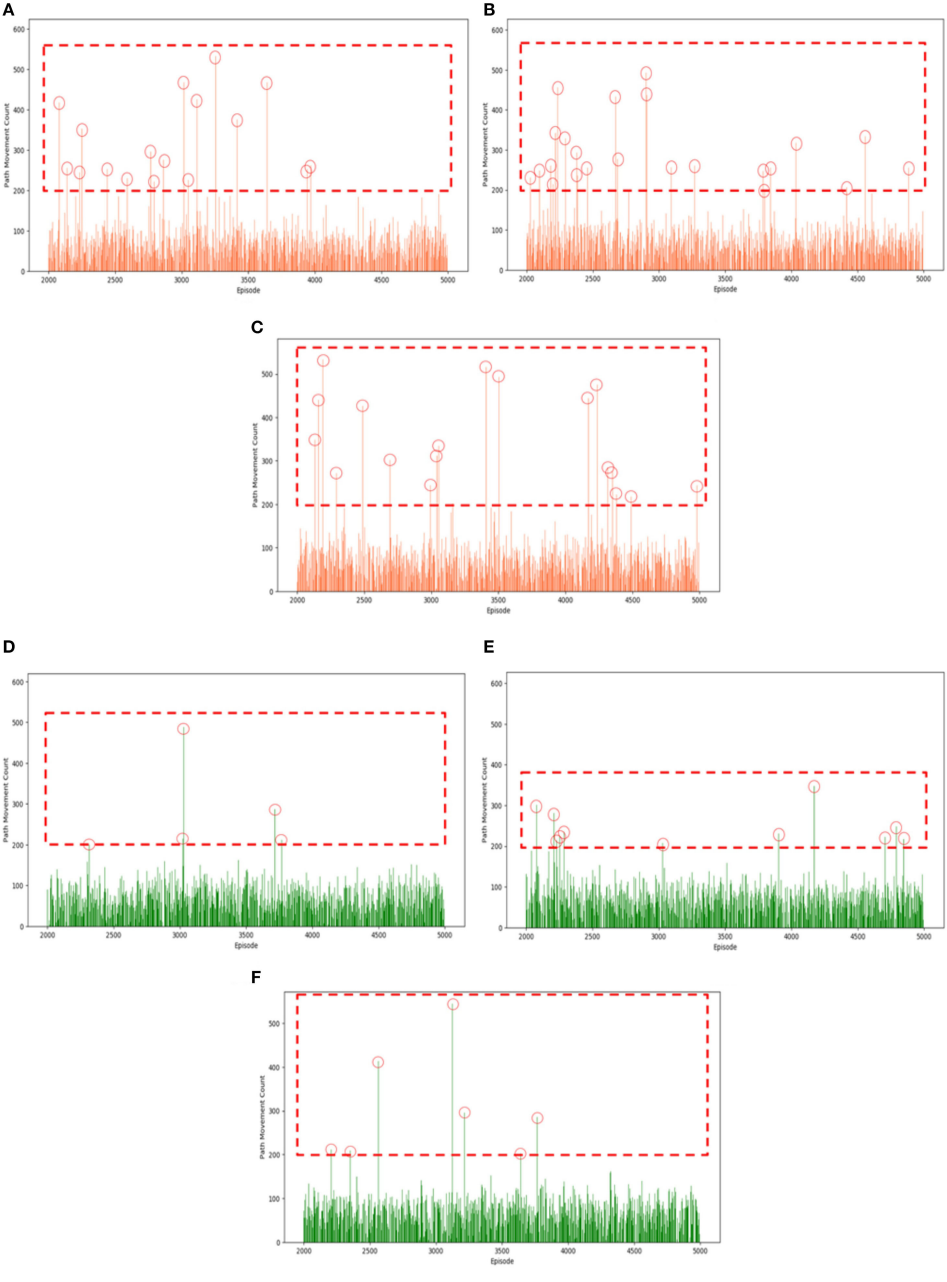
Status of intermittent path movement count increase problem in SAC algorithm: **(A)** 1st of Experiment #1; **(B)** 2nd of Experiment #1; **(C)** 3rd of Experiment #1; **(D)** 1st of Experiment #2; **(E)** 2nd of Experiment #2; **(F)** 3rd of Experiment #2.

Figure 12 shows a comparison of the SAC algorithm with and without the “Velocity Range-based Evaluation Method”, and the count of movements for every path found is plotted separately in a bar graph. In the bar graphs, the red circles within the red dashed area indicate that the count of path movement exceeds 200. For Experiment #1, this happened 17 times in the first trial, 23 times in the second trial, and 18 times in the third trial. The results of Experiment #2 were 5 times in the first trial, 11 times in the second trial, and 7 times in the third trial. In the case of path movement count with a large number, it is an inefficient result compared to a small number. When checking the case in which the count of the path movement exceeds 200, it was confirmed that the case where “Velocity Range-based Evaluation Method” was applied significantly reduced the count of the path movements that exceeded 200 compared to the case where it was not applied.

**Figure 13:**
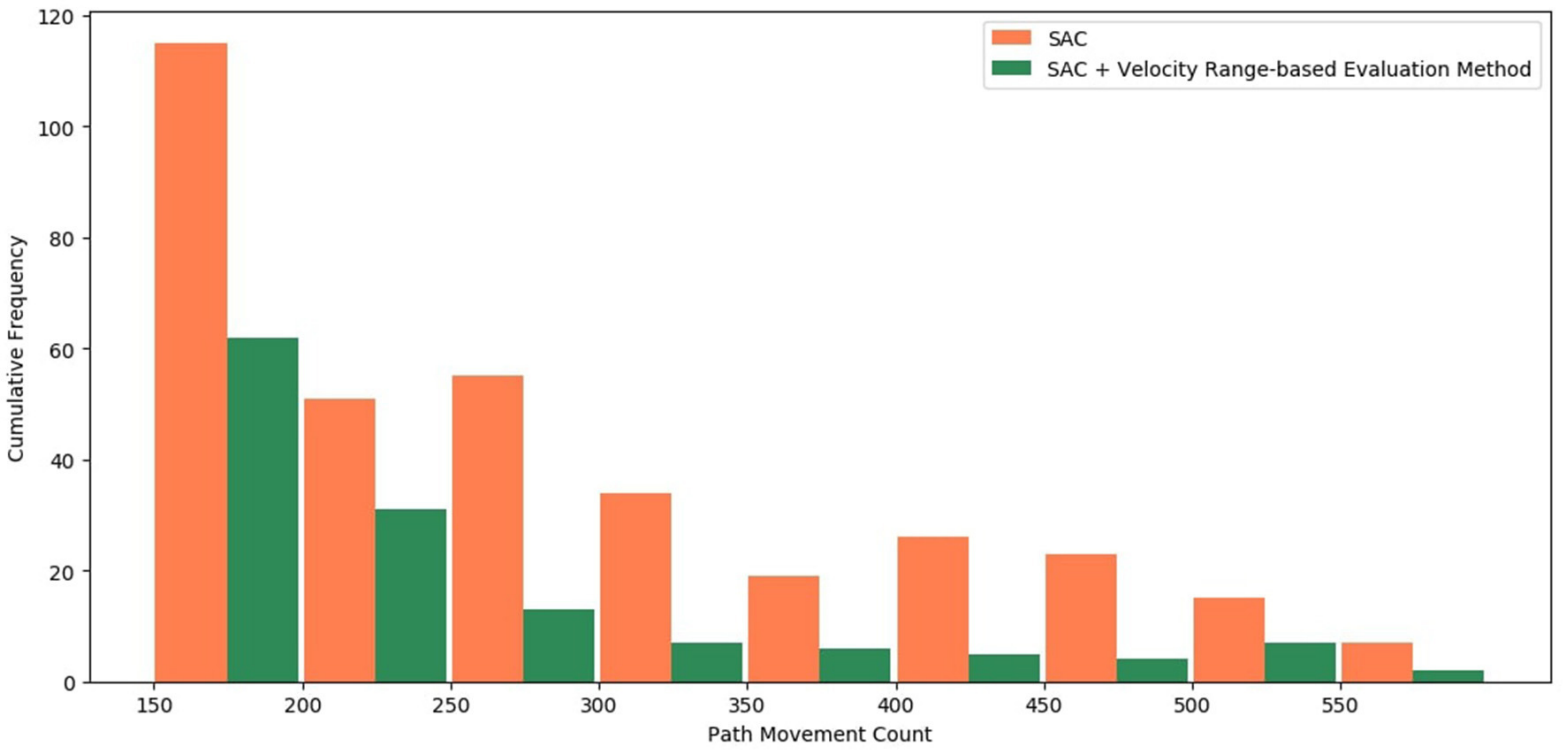
Comparison of cumulative frequency for intermittent path movement count increase problem.

A simple comparison can be made using the previous graphs, but it is necessary to check for a more quantitative and accurate comparison. The previous experimental results indicated that the count of path movement was concentrated at a value smaller than 150, and the cases the path movement count exceeds 200 is too few cases, so we lowered the threshold to 150 to check more data. It was thought that this phenomenon could be judged to be relatively inefficient if the count of path movement was >150, even if the episode was successful. Therefore, the counts of path movement are divided into nine ranges: (150–200), (200–250), (250–300), (300–350), (350–400), (400–450), (450–500), (500–550), and (550–600); the frequency of path movement count is checked for each range in Figure 13. For this review, the stable data of episodes 2,000 to 5,000 were checked, the case where the “Velocity Range-based Evaluation Method” was applied and the case where it was not applied were compared. The first experimental case is the case where the “Velocity Range-based Evaluation Method” was not applied and the color of the bar is marked in orange. The second experimental case is the case where the “Velocity Range-based Evaluation Method” was applied and the color of the bar is marked in green. Between episodes 2,000 and 5,000, the path movement counts exceeded 150 have occurred 345 times in the first case and 137 times in the second case. In the first case, it occurred 115 times in the 150–200 range, 51 times in the 200–250 range, 55 times in the 250–300 range, 34 times in the 300–350 range, 19 times in the 350–400 range, 26 times in the 400–450 range, 23 times in the 450–500 range, 15 times in the 500–550 range, and 7 times in the 550–600 range. In the second case, it occurred 62 times in the 150–200 range, 31 times in the 200–250 range, 13 times in the 250–300 range, 7 times in the 300–350 range, 6 times in the 350–400 range, 5 times in the 400–450 range, 4 times in the 450–500 range, 7 times in the 500–550 range, and 2 times in the 550–600 range. The frequency of the “Intermittent Path Movement Count Increase Problem” was reduced by about 66% and it is reduced up to 73% in the 250 to 600 range. Consequently, the proposed “Velocity Range-based Evaluation Method” is clearly effective in improving the “Intermittent Path Movement Count Increase Problem”.

**Figure 14:**
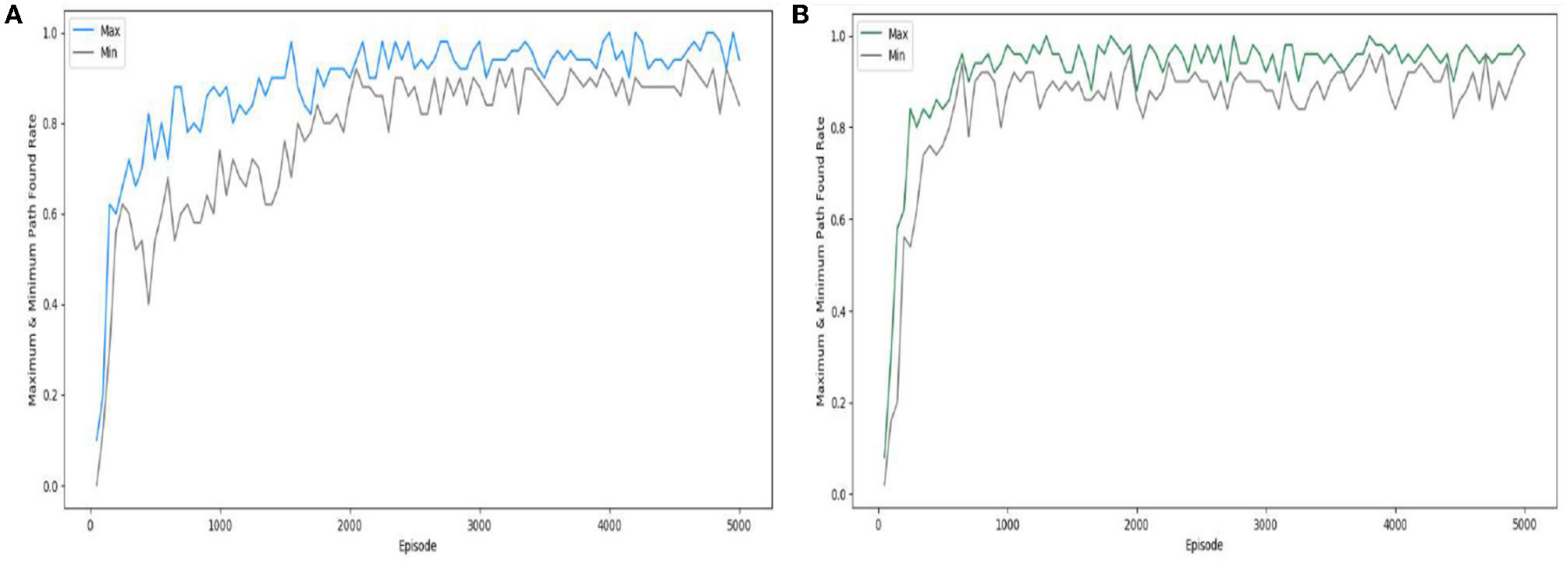
Comparison of the path founding rate: **(A)** SAC; **(B)** SAC with “Velocity Range-based Evaluation Method”.

Figure 14 shows the difference between the SAC algorithm experiment and the experiment with the “Velocity Range-based Evaluation Method” applied to the SAC algorithm, which was conducted three times each, by calculating the percentage of successfully finding the path every 50 episodes and visualizing the maximum and minimum rates. In the case of the SAC algorithm, the difference is quite large at the beginning of learning and gradually decreases as learning progresses, while in the case of the “Velocity Range-based Evaluation Method” applied to the SAC algorithm, we can see the feature that the experiment proceeds with little difference in deviation from the beginning of learning. This feature also has the advantage that the learning effect can be seen in a short time in the case of the “Velocity Range-based Evaluation Method” applied to the SAC algorithm. Therefore, the small performance difference between the experiments and the rapid and stable learning from the beginning are the advantages of the “Velocity Range-based Evaluation Method” method.

## 5. Conclusions

In this paper, experiments were conducted in a simulation environment using LiDAR sensors and deep reinforcement learning for the self-moving path planning of a single autonomous mobile robot in a small-scale warehouse environment, and additional performance improvements were proposed and verified. In constructing the experimental environment, global path planning was not considered, and local path planning was replaced with deep reinforcement learning. In terms of hardware, an autonomous mobile robot using 21 channels of LiDAR sensors was simulated. In particular, to learn path planning using reinforcement learning for an autonomous driving environment, it was necessary to consider a continuous action space that was different from those used in previous studies. For this reason, the deep reinforcement learning algorithm is a policy-based type of policy gradient algorithm. The DDPG algorithm and SAC algorithm were used. Moreover, the state, action, and reward methods were defined in consideration of the above-mentioned points. Further, to improve the additional performance and stability of reinforcement learning for autonomous driving, the “Velocity Range-based Evaluation Method” was proposed for the Reward technique. The experiment results were classified by the algorithm, and individual experiment results were confirmed. In addition, two types of graphs, a simple plot graph and a box plot graph, were shown to compare and analyze results. To capture the trend and efficiency of learning, we checked the change of final reward value and the movement count of the paths found, and the performance of the SAC algorithm was found to be better than the DDPG algorithm. The effectiveness of the proposed method “Velocity Range-based Evaluation Method” was confirmed by the fact that the final reward value was higher when it was applied than when it was not applied, and this result was also verified statistically. In addition, we also confirmed that the frequency of the phenomenon of intermittent increasing of the path movement count, which is the “Intermittent Path Movement Count Increase Problem” was reduced by about 66%. It reduces the difference between each experiment, enabling consistent learning and confirming fast learning performance with stable learning from the beginning. The “Velocity Range-based Evaluation Method” method can be used when implementing the autonomous driving of a mobile robot. To apply it, it is necessary to check and redefine the values of velocity range and evaluation score according to the experimental environment.

This research focuses on the implementation of autonomous mobile robots and the use of deep reinforcement learning to find the optimal path for autonomous driving. Autonomous mobile robots are rapidly being utilized in various fields and can be easily seen in our lives. Autonomous mobile robots that used to move around buildings are expected to evolve into delivery robots that deliver long distances in the near future, and autonomous robots for security surveillance. These autonomous mobile robots are expected to reduce costs, increase efficiency, and provide convenience to society and businesses. However, on the other hand, in terms of employment, there are disadvantages of replacing people's jobs with robots, and security concerns may be raised, such as the risk of exposing personal information collected through sensors and cameras. Despite these concerns, the utilization and application of robots is expected to expand rapidly. In particular, for the application of delivery robots and autonomous robots for surveillance, standards for authorization/permission must be established, relevant laws and regulations must be established, and inefficient regulations that hinder the use of robots must be actively relaxed.

As a future research direction, we would like to review the areas that need to be realistically identified and considered in order to use reinforcement learning for optimizing the path planning of mobile robots, develop additional evaluation criteria to strengthen the objective analysis and interpretation of experimental results, further strengthen the statistical interpretation to prove them, and solve the inherent problems and constraints in path planning based on reinforcement learning, and use reinforcement learning technology for actual mobile robots.

## Data availability statement

The original contributions presented in the study are included in the article/supplementary material, further inquiries can be directed to the corresponding author.

## Author contributions

All authors listed have made a substantial, direct, and intellectual contribution to the work and approved it for publication.
